# Ribosome biogenesis is a downstream effector of the oncogenic U2AF1-S34F mutation

**DOI:** 10.1371/journal.pbio.3000920

**Published:** 2020-11-02

**Authors:** Abdalla Akef, Kathy McGraw, Steven D. Cappell, Daniel R. Larson

**Affiliations:** 1 Laboratory of Receptor Biology and Gene Expression, National Cancer Institute, National Institutes of Health, Bethesda, Maryland, United States of America; 2 Department of Malignant Hematology, Moffitt Cancer Center, Tampa, Florida, United States of America; 3 Laboratory of Cancer Biology and Genetics, National Cancer Institute, National Institutes of Health, Bethesda, Maryland, United States of America; Yale University, UNITED STATES

## Abstract

U2 Small Nuclear RNA Auxiliary Factor 1 (U2AF1) forms a heterodimeric complex with U2AF2 that is primarily responsible for 3ʹ splice site selection. U2AF1 mutations have been identified in most cancers but are prevalent in Myelodysplastic Syndrome (MDS) and Acute Myeloid Leukemia (AML), and the most common mutation is a missense substitution of serine-34 to phenylalanine (S34F). The U2AF heterodimer also has a noncanonical function as a translational regulator. Here, we report that the U2AF1-S34F mutation results in specific misregulation of the translation initiation and ribosome biogenesis machinery. The net result is an increase in mRNA translation at the single-cell level. Among the translationally up-regulated targets of U2AF1-S34F is Nucleophosmin 1 (NPM1), which is a major driver of myeloid malignancy. Depletion of NPM1 impairs the viability of the U2AF1-S34F mutant cells and causes ribosomal RNA (rRNA) processing defects, thus indicating an unanticipated synthetic interaction between U2AF1, NPM1, and ribosome biogenesis. Our results establish a unique molecular phenotype for the U2AF1 mutation that recapitulates translational misregulation in myeloid disease.

## Abstract 2

This study reveals a novel, to our knowledge, connection between the splice factor U2AF1 mutation responsible for myelodysplastic syndrome and ribosome biogenesis. Silencing the expression of the ribosome biogenesis factor NPM1 alters the U2AF1-S34F mutant cell proteome, down-regulating ribosome biogenesis and cell cycle progression factors.

## Introduction

U2 Small Nuclear RNA Auxiliary Factor 1 (U2AF1; also known as U2AF35) forms a heterodimeric complex with U2AF2 (U2AF65) that is primarily responsible for 3ʹ splice site selection [1]. Several U2AF1 mutations have been identified in Myelodysplastic Syndrome (MDS) and Lung Adenocarcinoma (LUAD) patients [2], the most common of which is a single missense substitution of serine-34 to phenylalanine (S34F). The mutation is heterozygous, occurs early in disease progression, and is a hotspot mutation in the RNA binding domain of the protein, strongly suggesting a gain-of-function, oncogenic mutation. Although several groups have examined alternative splicing alterations that are mediated by the S34F mutation [2–5], there is no clear set of splicing events that can explain the tumorigenic role of the S34F mutation. Previously, we reported that U2AF1, in complex with U2AF2, plays a noncanonical role in translation regulation through direct interactions with target RNA in the cytosol [6]. Recently, a U2AF1 paralog, U2AF26, has also been shown to regulate the translation of its bound mRNAs [7]. Using RNP immunoprecipitation coupled to sequencing (RIP-seq) and polysome profiling, we found that the S34F mutation resulted in loss of binding and a concomitant increase in the translation of these target mRNAs. These mRNA targets were enriched for components of the mRNA translation and ribosome biogenesis machinery [6]. However, it is unknown how ribosome biogenesis and translational misregulation play a role in the physiology of U2AF1 mutations and contribute to leukemogenesis.

Intriguingly, changes in translational regulation and ribosome biogenesis are hallmarks of myeloid diseases. Hematopoietic stem cells maintain a quiescent state by limiting protein synthesis rates, and altering this balance leads to defects in cellular differentiation and lineage commitment [8]. In line with this observation, germ-line mutations resulting in haploinsuffiency of ribosomal protein large and small subunits (RPL and RPS, respectively) such as *RPS19*, *RPS26*, *RPS17*, *RPL5*, and *RPL11* or assembly chaperones such as TSR2 ribosome maturation factor (*TSR2*) result in “ribosomopathies,” which preferentially lead to defects in myeloid differentiation and present as anemias such as Diamond-Blackfan Anemia and Shwachman-Diamond syndrome [9]. Similarly, recent evidence suggests that MDS is characterized by increased protein synthesis in the population of cluster of differentiation (CD)123+ MDS stem cells from human patients [10]. These malignant MDS stem cells show increased translation yet retain a quiescent phenotype and are predominantly in the G0 phase of the cell cycle. Finally, therapeutics that target RNA polymerase I, the polymerase responsible for synthesizing rRNA, have shown specific activity against leukemia initiating cells in Acute Myeloid Leukemia (AML) [11]. In total, these studies suggest that changes in translation and ribosome biogenesis contribute to the pathology of myeloid disease and are therapeutic targets. Yet in many cases, especially for diseases such as MDS and AML that arise from somatic mutations in stem and progenitor cells, the molecular pathways have not been described.

Among the top translationally up-regulated targets of U2AF1-S34F is Nucleophosmin 1 (*NPM1*) [6], which plays a functional role in ribosome biogenesis and is mutated in a fifth of all AML cases. NPM1 (also known as B23) is a nucleic acid binding protein involved in the processing and nucleocytoplasmic transport of ribosomal RNA (rRNA) [12,13], [14]. Yet, despite the involvement of NPM1 in such a crucial process as ribosome biogenesis, fibroblasts derived from an *NPM1*^*−/−*^ mouse could survive in culture with modest defects [15]. Moreover, the expression of a dominant-negative NPM1 mutant did not cause noticeable defects in the steady state levels of the 28S and 18S rRNA [14]. Furthermore, a previous study that employed a small interfering RNA (siRNA) screen against nucleolar factors showed that NPM1 silencing did not cause major defects in rRNA processing [16]. These findings indicate that NPM1 is dispensable for cell viability. The AML-linked mutations cause the mislocalization of NPM1 to the cytoplasm [17] potentially leading to an imbalance of nucleolar and cytoplasmic functions, including changes in gene expression [18] and protein 53 (p53) activity [19–21]. Strikingly, NPM1 mutations rarely occur outside of myeloid malignancies (i.e., MDS and AML), suggesting that NPM1 plays a unique role in the differentiation and/or proliferation of myeloid cells. However, the role of NPM1 in promoting myeloid malignancies remains incompletely understood. For example, the established role that NPM1 plays in ribosome biogenesis has not been definitively linked to disease phenotypes. Moreover, despite the pervasiveness of splicing factor and NPM1 mutations in myeloid disease, there is no reported functional connection between these factors.

Here, we establish a direct link between 2 major drivers of myeloid disease: U2AF1 and NPM1. We find that a heterozygous U2AF1-S34F mutation leads to translational reprogramming of specific subsets of mRNAs in both immortalized epithelial cells and mouse-derived common myeloid progenitors, similar to that observed in MDS patients. Moreover, cells with the S34F mutation are dependent on NPM1 for cell cycle progression. Depletion of NPM1 impaired the viability of the S34F-harboring cells, but not wild-type (wt)/wt cells. wt/S34F cells with reduced NPM1 displayed 1) lower proliferative capacity, 2) accumulation in the S phase of the cell cycle, and 3) specific rRNA processing defects. A quantitative proteomic analysis indicated that ribosome biogenesis factors and cell cycle DNA replication factors were significantly down-regulated in wt/S34F cells depleted of NPM1. Our results demonstrate that NPM1 is a downstream effector of the oncogenic changes mediated by the U2AF1-S34F mutation. Moreover, this viability defect explains why U2AF1 and NPM1 mutations are mutually exclusive in MDS and AML patients. These data indicate an unanticipated functional connection between a splicing factor mutation and the ribosome biogenesis machinery.

## Results

### The U2AF1-S34F mutation alters the level of translation initiation factors

Using RIP-seq, we previously reported that U2AF1 binds mRNA in the cytoplasm enriched for functional Gene Ontology (GO) categories that include “translation initiation” and “peptide biosynthetic process.” Here, we systematically address the changes in translational efficiency and total protein for those mRNAs using quantitative analysis of polysome profiles and mass spectrometry. To dissect the molecular mechanisms perturbed by the U2AF1-S34F mutation, we employed 3 human bronchial epithelial cell (HBEC) lines [22] that were generated by gene editing: 1) biallelic wt U2AF1 (herein referred to as wt/wt), 2) wt/U2AF1-S34F heterozygous (wt/S34F), and 3) a frameshift of the U2AF1-S34F allele (wt/S34F-) as previously described [3,6].

**Fig 1:**
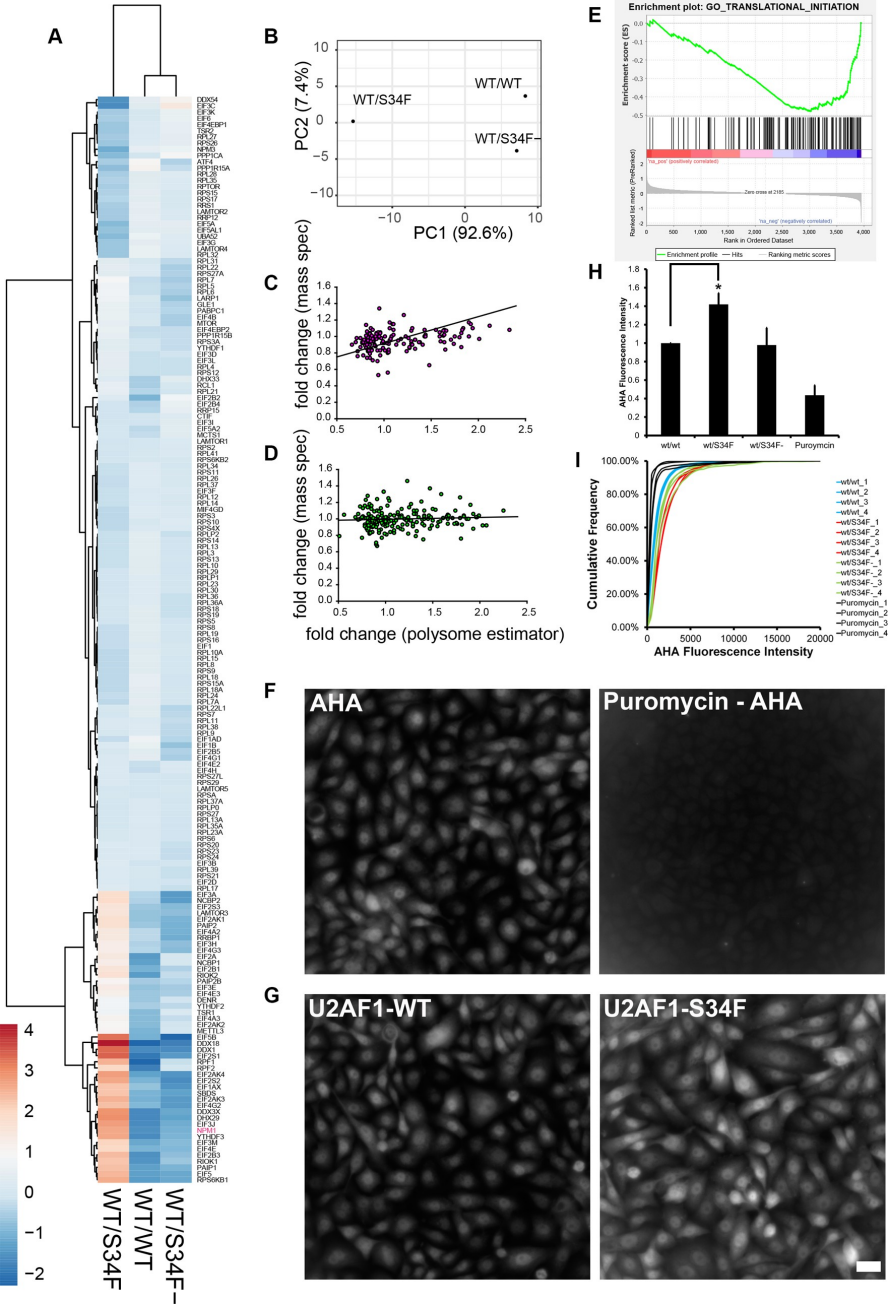
The U2AF1-S34F mutation up-regulates a subset of translation and ribosome biogenesis factors and increases nascent polypeptide production. (A) Heat map and hierarchical clustering of the weighted estimator applied to RNA-seq from polysome fractions. The genes are the 175 translation initiation genes present in all 8 fractions of each sample (wt/wt, wt/S34F, wt/S34F-) cells (24 total measurements). Rows are centered; no scaling is applied to rows. Rows are clustered using Euclidean distance and Ward linkage. Columns are clustered using correlation distance and average linkage. 175 rows, 3 columns. The underlying data are in S1 Table. (B) Principal component analysis of weighted estimator applied to RNA-seq from polysome fractions. No scaling is applied to rows; SVD with imputation is used to calculate principal components. *X* and *Y* axis show PC 1 and PC 2, which explain 92.6% and 7.4% of the total variance, respectively. *N* = 3 data points. The underlying data are in S1 Table. (C) Comparison of FC for polysome measurement to mass spectrometry measurements for genes/proteins in the “translation initiation” GO category (152 genes, S2 Table). FC is wt/S34F over wt/wt. Regression line is reduced major axis regression with slope = 0.32 ± 0.17, intercept = 0.59 ± 0.04. The underlying data are in S2 Table. (D) Comparison of FC for polysome measurement to mass spectrometry measurements for genes/proteins with similar abundance to proteins in Fig 1C. FC is wt/S34F over wt/wt. Regression line is reduced major axis regression with slope = 0.04 ± 0.04, intercept = 0.98 ± 0.10. The underlying data are in S2 Table. (E) GSEA based on MS data. Analysis was carried out on preranked lists using GSEA 4.0.3. Translation initiation is negatively enriched in wt/S34F cells compared with wt/wt cells. Normalized enrichment score = −2.20. FDR q-value = 0.008. FWER *p*-value = 0.027. Full analysis is shown in S3 Fig. The underlying data are in S2 Table. (F) Puromycin treatment abrogates the nascent polypeptide fluorescence signal. Scale bar = 10 μm. (G) The U2AF1-S34F mutation up-regulates nascent polypeptide production. Scale bar = 10 μm. (H) Total cellular fluorescence intensity was quantified. Each bar represents the average and standard error of 4 independent experiments. The underlying data are in S1 Data. (I) Cumulative fluorescence intensity of the AHA signal. The underlying data are in S1 Data. AHA, azido-homoalanine; ATF4, activating transcription factor 4; CTIF, cap = binding-complex–dependent translation initiation factor; DDX, DEAD-box helicase; DENR, density = regulated reinitiation and release factor; DHX, DEAH-box helicase; EIF, eukaryotic initiation factor; FC, fold change; FDR, false discovery rate; FWER, family-wise error rate; GLE1, GLFG lethal 1; GO, Gene Ontology; GSEA, gene set enrichment analysis; LAMTOR, late endosomal/lysosomal adaptor, MAPK and MTOR activator; LARP1, La ribonucleoprotein 1, translational regulator; MCTS1, Malignant T-cell–amplified sequence 1 reinitiation and release factor; METTL3, methyltransferase-like 3; MIF4GD, MIF4G domain containing; MS, mass spectrometry; MTOR, mechanistic target of rapamycin kinase; NCBP, nuclear cap binding protein; NPM1, Nucleophosmin 1; PABPC1, poly(A) binding protein cytoplasmic 1; PAIP2, poly(A) binding protein interacting protein 2; PC, principal component; PPP1CA, protein phosphatase 1 catalytic subunit alpha; RCL1, RNA terminal phosphate cyclase like-1; RIOK1, RIO kinase 1; RNA-seq, RNA sequencing; RPL, ribosomal protein large subunit; RPLP2, ribosomal protein lateral stalk subunit P2; RPS, ribosomal protein small subunit; RPSA, ribosomal protein SA; RPTOR, regulatory associated protein of MTOR complex 1; RRBP1, ribosome binding protein 1; RRP, ribosomal RNA processing 1; RRS, ribosome biogenesis regulator 1 homolog; SBDS, Shwachman-Bodian-Diamond syndrome ribosome maturation factor; SVD, singular value decomposition; S34F, serine-34 to phenylalanine substitution; TSR1/2, TSR 1/2 ribosome maturation factors; UBA52, ubiquitin A-52 residue ribosomal protein fusion product 1; U2AF1, U2 Small Nuclear RNA Auxiliar Factor 1; wt, wild-type; YTHDF, YTH N6-methyladenosine RNA binding protein.

By analyzing polysome profiling data from wt/wt, wt/S34F, and wt/S34F- cells (previously published in [6]), we sought to determine whether the S34F mutation altered the translational regulation of mRNA in the GO category “translation initiation” (GO:0006413). This category consists of 185 genes, overlaps many of the other categories we observed as U2AF1-bound in RIP-seq, and contains ribosomal subunits, eukaryotic initiation factors, and some ribosome biogenesis factors. Our analysis is based on a weighted estimator of translation efficiency as determined by RNA abundance in the polysome profile. Briefly, this analysis attempts to infer the number of ribosomes on an mRNA by comparing the quantitative polysome absorbance profile, the normalized counts in each fraction from RNA sequencing (RNA-seq), and the theoretical expectation for the sedimentation velocity. We applied this estimator for 20,997 RNAs that were present in each fraction of each sample in our polysome analysis (24 total measurements, S1 Table). Out of 185 mRNA targets in the translation initiation GO category, 175 met these criteria, and we performed principal component analysis and hierarchical clustering on the translational efficiency computed from this estimator (Fig 1A and 1B). We also carried out the identical analysis using a simpler estimator based only on polysome/monosome ratio (S1 Fig). These data indicate that the S34F mutation results in widespread changes in translation efficiency in mRNA in the GO category of translation initiation. However, these changes are almost completely rescued by frameshifting the mutant allele, as indicated by the observation that the S34F mutation accounts for >90% of the variation in the samples (Fig 1B). Translation efficiency both increases and decreases for genes in this family, with 2 distinct subgroups evident in the clustering analysis. Increases in translation efficiency occur for a number of eukaryotic initiation factors (*EIF5*, *EIF4E*, *EIF3E*, *EIF3H*, *EIF3J*, *EIF3M*), DEAD-box (DDX) and DEAH-box (DHX) helicases (*DDX18*, *DDX1*, *DDX3X*, *DHX29*), and *NPM1*. Decreases in efficiency occur for some negative regulators (*EIF4EBP1*), members of the mechanistic target of rapamycin kinase (mTOR) pathway (regulatory associated protein of MTOR complex 1 [*RPTOR*]; late endosomal/lysosomal adaptor, MAPK and MTOR activators [*LAMTOR2* and *LAMTOR4*]), and a subset of ribosomal subunits. Notably, ribosomal subunits were either unchanged or decreased, and several genes implicated in Diamond-Blackfan Anemia (TSR2 ribosome maturation factor [*TSR2*], *RPS26*, *RPS17*) clustered in the group showing the largest decreases in translation efficiency. We also plotted mean polysome profiles for the top, middle, and bottom quintiles to demonstrate the extent of the changes in translational efficiency (S2 Fig). Thus, a single amino acid change in the splicing factor U2AF1 results in changes in translation efficiency across messages coding for proteins that are also involved in translation initiation.

To independently interrogate changes in protein levels resulting from the S34F mutation, we used quantitative mass spectrometry (MS). wt/wt and wt/S34F cells were harvested and lysed, and total protein was quantified and digested. The peptides were conjugated to tandem mass tags (TMT), and MS was performed as previously described [23,24]. We detected 4,167 proteins present in 3 replicates of isobaric mass tag-MS in wt/wt and wt/S34F cells (6 total measurements). Comparing the fold change (FC) measured from MS to the FC measured from polysome profiling across all proteins that are above the threshold in both studies (3,953 proteins, S2 Table) revealed little to no correlation between the 2 measures. The difficulty in comparing translation efficiency as determined through sequencing approaches and MS has been reported previously and can be due to technical artifacts and also the relative contributions of protein synthesis and decay in determining total protein abundance [25]. Nevertheless, for the translation initiation GO category (152 genes above threshold in polysome and MS measurements), there was a linear relationship between polysome FC and MS FC (Fig 1C and S2 Table). Using reduced major axis regression, which effectively handles errors in both the *x*- and *y*-variable [26], gives a regression coefficient of 0.32 ± 0.17. This slope indicates that changes in translation efficiency are in fact resulting in changes in protein abundance, albeit with a smaller dynamic range. In contrast, a randomly selected group of proteins with similar abundance shows no correlation between polysome FC and MS FC (Fig 1D, regression coefficient = 0.04).

Next, we used our MS data in wt/wt and wt/S34F as a more faithful measure of gene expression changes that arise through the S34F mutation. Because changes in translation efficiency and/or protein abundance would not be reflected in RNA abundance or isoform changes measured through RNA-seq, we performed gene set enrichment analysis (GSEA) on protein abundance [27]. Positive enrichment (proteins that are increased in the wt/S34F cells compared with wt/wt cells) did not result in any gene sets with a false discovery rate (FDR) < 0.2 or family-wise error rate (FWER) < 0.2. However, analyzing the negatively enriched gene sets (proteins that are decreased in the wt/S34F cells compared with the wt/wt cells) uniquely identified genes/proteins involved in translation initiation (FDR = 0.008, FWER = 0.027, normalized enrichment score = −2.20) (Fig 1E and S3 Fig). Thus, MS identifies changes in translation initiation proteins as the dominant molecular phenotype for the U2AF1-S34F mutation.

Finally, we sought to examine the functional consequences of misregulation of the translation initiation machinery at the single-cell level. Our genome-wide assays pointed in multiple directions; translation initiation as a category was negatively enriched in wt/S34F cells compared with wt/wt cells, but this category encompasses both positive and negative regulators of initiation. To measure the integrated output of these changes, we compared mRNA translation rates between the wt/wt and the wt/S34F cells using fluorescent noncanonical amino acid tagging (FUNCAT) as previously described [28]. Cells were pulsed with the methionine analog azido-homoalanine (AHA) for 1 hour. Subsequently, cells were fixed and permeabilized, and the AHA-containing nascent polypeptides were labeled for microscopy using click chemistry. First, we confirmed that puromycin treatment abolished the AHA fluorescence signal (Fig 1F). Next, we found that the S34F mutation caused an up-regulation in translation (Fig 1G, quantification in Fig 1H). Comparing fluorescence intensity at the single-cell level indicated that the elevated level of mRNA translation in the wt/S34F cells was representative of the whole cell population rather than a few outlier cells (Fig 1I). Taken together, we conclude that the misregulation of the translation initiation machinery in wt/S34F cells results in a net increase in mRNA translation.

Because MS reports primarily on relative abundance changes and AHA labeling measures amino acid incorporation rates, we sought an intermediate method that would report on the nature of the proteins being up-regulated in wt/S34F cells. We probed for nascent polypeptides using puromycin labeling as described in the previously published SUnSet protocol [29]. This approach involves treating cells with puromycin for 1 hour. Puromycin is incorporated into nascent polypeptides and the nascent polypeptides are detected by western blot using an anti-puromycin antibody. Puromycin labeling showed higher levels of nascent polypeptides in wt/S34F cells over wt/wt cells (S4 Fig), confirming our single-cell AHA labeling. Surprisingly, the wt/S34F cellular proteome had a different mobility in the gel in comparison to wt/wt cells, suggesting widespread proteomic alterations due to the S34F mutation. Thus, increased translation in wt/S34F is not simply an amplification of the translation program in wt/wt cells.

In summary, a single missense mutation in U2AF1 results in widespread changes to translation as measured both by polysome sequencing and mass spectrometry. Proteins involved in translation initiation—including eukaryotic initiation factors, ribosome subunits, positive and negative translational regulators, and ribosome biogenesis factors—are significantly affected. Despite a decrease in protein abundance for the GO category of translation initiation as a whole in wt/S34F cells compared with wt/wt cells, protein synthesis is elevated in these cells, recapitulating the phenotype seen in human MDS stem cells [10].

### wt/S34F cells require NPM1 for survival in a p53-independent manner

**Fig 2:**
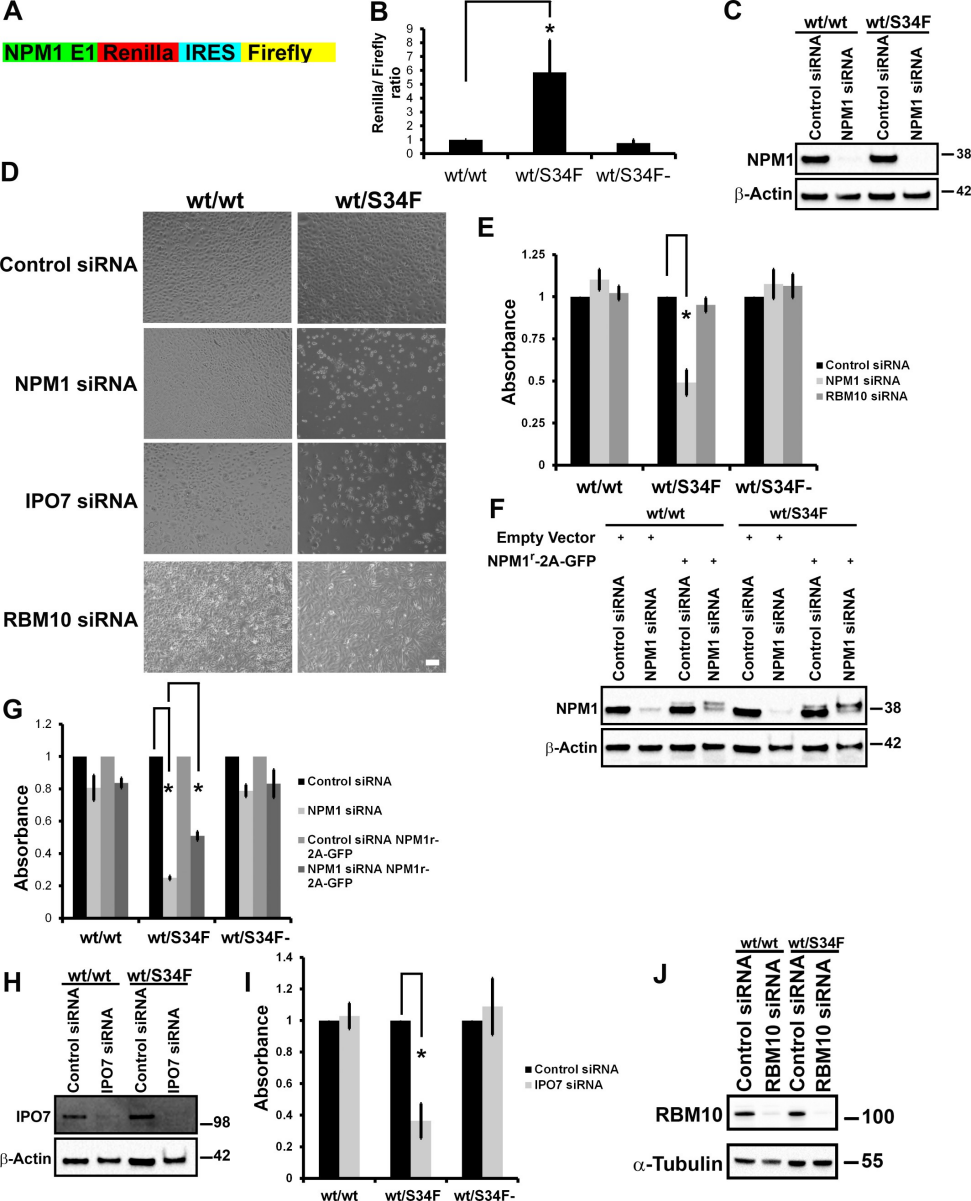
NPM1 and IPO7 are required for the viability of the wt/S34F cells. (A) A schematic diagram of the *NPM1-luciferase* reporter. The reporter consists of NPM1 exon 1, *Renilla* luciferase, CrPV IRES, and Firefly luciferase. (B) Normalized *Renilla* to Firefly luciferase activity demonstrates elevated translation levels of the *NPM1-luciferase* reporter. *p < 0.05 using a paired 2-sided *t* test. (C) Cells treated with siRNA directed against NPM1 were lysed, and proteins were separated on SDS-PAGE and analyzed by immunoblot using antibodies against NPM1 and α-tubulin. (D) Cells treated with the indicated siRNAs were imaged 144 hours post-siRNA transfection. Scale bar = 50 μm. (E) Quantification of cell viability using the WST-1 reagent. Each bar represents the average and standard error of at least 3 independent experiments. *p < 0.05 using a paired 2-sided *t* test. (F) Cells that express either NPM1^r^-2A-GFP or empty vector were treated with siRNA directed against NPM1 and were lysed, and proteins were separated on SDS-PAGE and analyzed by immunoblot using antibodies against NPM1 and β-actin. (G) Quantification of cell viability using the WST-1 reagent. Each bar represents the average and standard error of at least 3 independent experiments. *p < 0.05 using a paired 2-sided *t* test. (H) Cells treated with siRNA directed against IPO7 were lysed, and proteins were separated on SDS-PAGE and analyzed by immunoblot using antibodies against IPO7 and β-actin. (I) Quantification of cell viability using the WST-1 reagent. Each bar represents the average and standard error of 3 independent experiments. *p < 0.05 using a paired 2-sided *t* test. (J) Cells treated with siRNA directed against RBM10 were lysed, and proteins were separated on SDS-PAGE and analyzed by immunoblot using antibodies against RBM10 and β-actin. The underlying data are in S1 Data. E1, exon 1; GFP, green fluorescent protein; IPO7, Importin 7; IRES, Internal Ribosome Entry Site; NPM1: Nucleophosmin 1; RBM10, RNA binding motif protein 10; siRNA, small interfering RNA; S34F, serine-34 to phenylalanine substitution; WST-1, water-soluble tetrazolium-1; wt, wild-type.

Because NPM1 was identified first as a target of U2AF1 binding in the cytosol, is included in the translation initiation gene set, shows concordant increase in translational efficiency by polysome sequencing and protein abundance by MS (S2 Table), and is frequently altered in myeloid malignancy, we chose to focus on this mRNA/protein for in depth characterization. We first confirmed the up-regulation of NPM1 translation in wt/S34F cells using a dual-luciferase reporter. The first exon of *NPM1* was fused upstream to *Renilla* luciferase. To normalize for differences in cell number and copy number, the reporter also included a Firefly luciferase downstream of an Internal Ribosome Entry Site (IRES) sequence derived from the Cricket Paralysis Virus (CrPV) [30,31] (Fig 2A). The wt/S34F cells had an 5-fold higher *Renilla* to Firefly luciferase activity than wt/wt or wt/S34F- cells (Fig 2B), further confirming the up-regulation of NPM1 in cells harboring the U2AF1-S34F mutation and additionally indicating that the 5ʹ end of the mRNA is sufficient for increased translation. All cell lines had comparable luciferase reporter mRNA levels using reverse transcription–quantitative PCR (RT-qPCR) (S5 Fig).

We sought to examine whether increased NPM1 translation was a downstream mediator of the pro-tumorigenic phenotype of the U2AF1-S34F mutation. NPM1 was depleted by RNA interference (RNAi) in wt/wt, wt/S34F, and wt/S34F- cells (Fig 2C). We noticed a marked decrease in the viability of the wt/S34F cells upon NPM1 depletion (Fig 2D). No defects were observed for the wt/wt or wt/S34F- cells (Fig 2D and 2E). To quantitatively assay cell viability, we employed the water-soluble tetrazolium-1 (WST-1) assay, in which tetrazolium salt is added to the cells. Mitochondrial dehydrogenases cleave the tetrazolium salts to formazan, and the level of formazan is measured colorimetrically. A higher number of cells is accompanied by a higher dehydrogenase activity and more formazan salt is formed. We confirmed the decreased viability of wt/S34F cells depleted of NPM1 using the WST-1 assay (Fig 2E). We generated cell lines that express an RNAi-resistant NPM1 construct (NPM1^r^-2A-green fluorescent protein [GFP]) using lentiviral-mediated integration. A slower mobility band was detected for NPM1^r^-2A-GFP, which was unaffected by NPM1 siRNA treatment (Fig 2F). The viability of the wt/S34F cells that express NPM1^r^-2A-GFP was partially restored (Fig 2G). The partial rescue is likely due to the low expression levels of NPM1^r^-2A-GFP in comparison to endogenous NPM1 levels (Fig 2F).

We sought to corroborate these findings by depletion of a second ribosome biogenesis factor, Importin 7 (IPO7). IPO7 mRNA is also a direct U2AF1 target in the cytoplasm [6] and is responsible for the import of the large ribosomal subunit proteins RPL5 and RPL11 to the nucleus [32]. As for NPM1, IPO7 silencing by RNAi was also not reported to cause cell death [32]. Yet, IPO7 depletion also specifically impaired the survival of wt/S34F, but not wt/wt, cells (Fig 2D, 2H and 2I). Neither a scrambled control siRNA nor siRNA to RBM10, which is a splicing factor in the nucleus, had any effect on cell viability in any cell line (Fig 2D, 2E and 2J). Taken together, NPM1 and IPO7, both targets of U2AF1 binding and translational regulation (S1 Table) and both involved in transport and/or ribosome biogenesis, are essential for viability in the wt/S34F cells, but not in the wt/wt cells.

**Fig 3:**
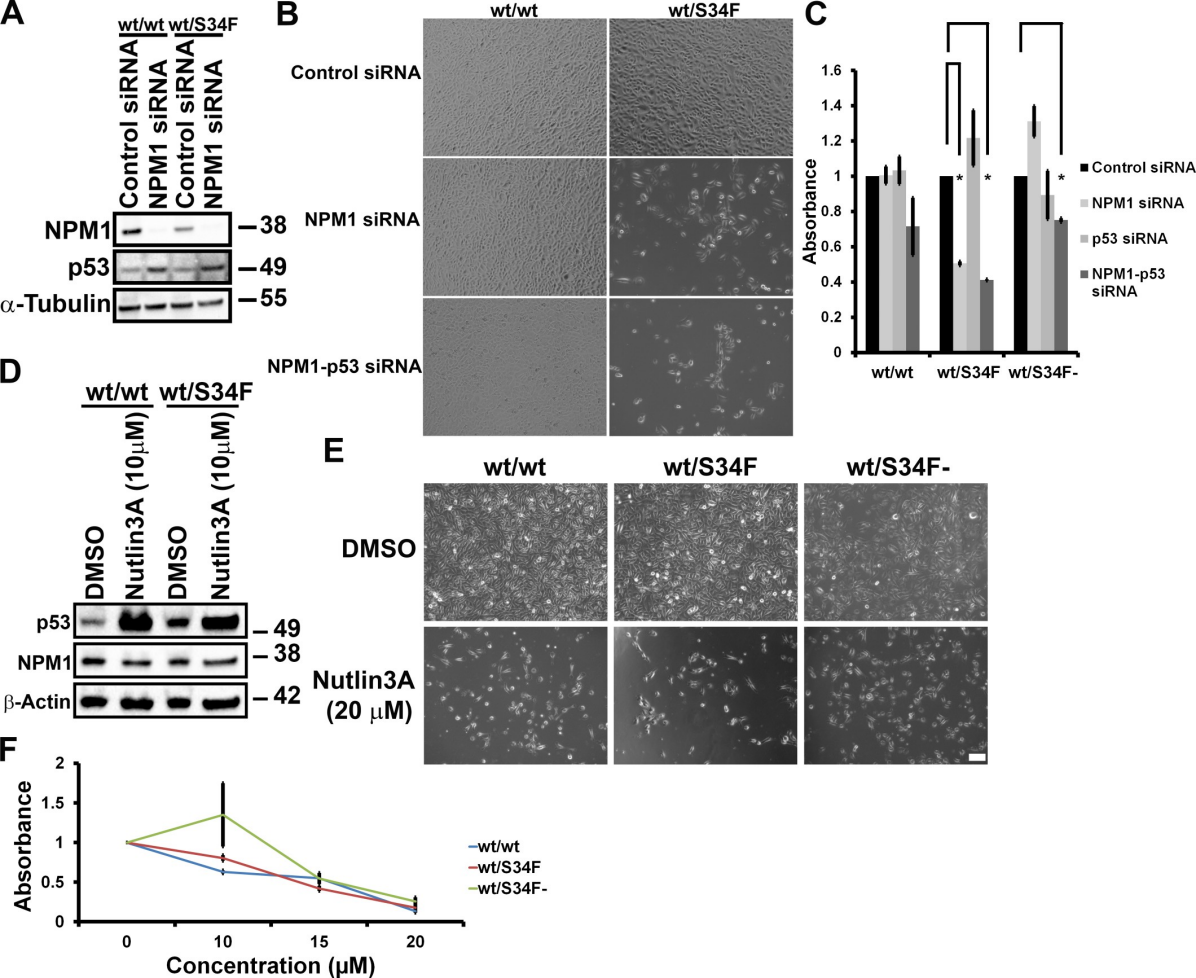
The requirement of wt/S34F cells for NPM1 is independent of p53. (A) Cells treated with siRNA directed against NPM1 were lysed and proteins separated on SDS-PAGE and analyzed by immunoblot using antibodies against NPM1, p53, and α-tubulin. (B) Cells treated with the indicated siRNAs were imaged 144 hours post-siRNA transfection. Scale bar = 50 μm. (C) Quantification of cell viability using the WST-1 reagent. Each bar represents the average and standard error of 3 independent experiments. *p < 0.05 using a paired 2-sided *t* test. (D) Cells treated with 10 μM Nutlin3A for 36 hours were lysed, and proteins were separated on SDS-PAGE and analyzed by immunoblot using antibodies against NPM1, p53, and β-actin. (E) Nutlin3A treatment impairs the viability of wt/wt, wt/S34F, and wt/S34F- cells. Scale bar = 50 μm. (F) Cell viability was assessed 36 hours after treatment with the indicated doses of Nutlin3A using the WST-1 reagent. The underlying data are in S1 Data. DMSO, dimethyl sulfoxide; NPM1, Nucleophosmin 1; p53: protein 53; siRNA, small interfering RNA; S34F, serine-34 to phenylalanine substitution; WST-1, water-soluble tetrazolium-1; wt, wild-type.

It is known that disruption of ribosome biogenesis and/or alteration of the stoichiometry of ribosome subunits can trigger a p53 response in cells [33,34]. Specifically, NPM1 down-regulates the activity of p53 by sequestering the Alternate Reading Frame (ARF, also known as human p14) in the nucleolus and preventing the ARF–mouse 3T3 cell double minute 2 proto-oncogene (MDM2) interaction, thus enabling MDM2-mediated degradation of p53 [19,35,36]. We therefore investigated whether the viability defect of the wt/S34F cells depleted of NPM1 was dependent on p53 activity. First, we compared p53 levels between wt/wt and wt/S34F cells depleted of NPM1. NPM1 depletion caused the up-regulation of p53 in both the wt/wt and wt/S34F cells to a comparable extent (Fig 3A). Moreover, the synergistic depletion of NPM1 and p53 did not rescue the viability defect of the wt/S34F cells caused by the absence of NPM1 (Fig 3B and 3C). Next, we sought to assess whether increasing p53 levels in an NPM1-independent mechanism would specifically impair the viability of the wt/S34F cells. p53 levels were induced by treating cells with 10 μM Nutlin3A [37] (Fig 3D). p53 activation by increasing Nutlin3A concentration impaired the viability of wt/wt, wt/S34F, and wt/S34F- cells to a similar extent (Fig 3E, quantification in Fig 3F). Thus, we conclude that the depletion of NPM1 impairs the viability of the wt/S34F cells through a p53-independent pathway.

### wt/S34F cells depleted of NPM1 exhibit cycle progression defects and changes in ribosome biogenesis factors

**Fig 4:**
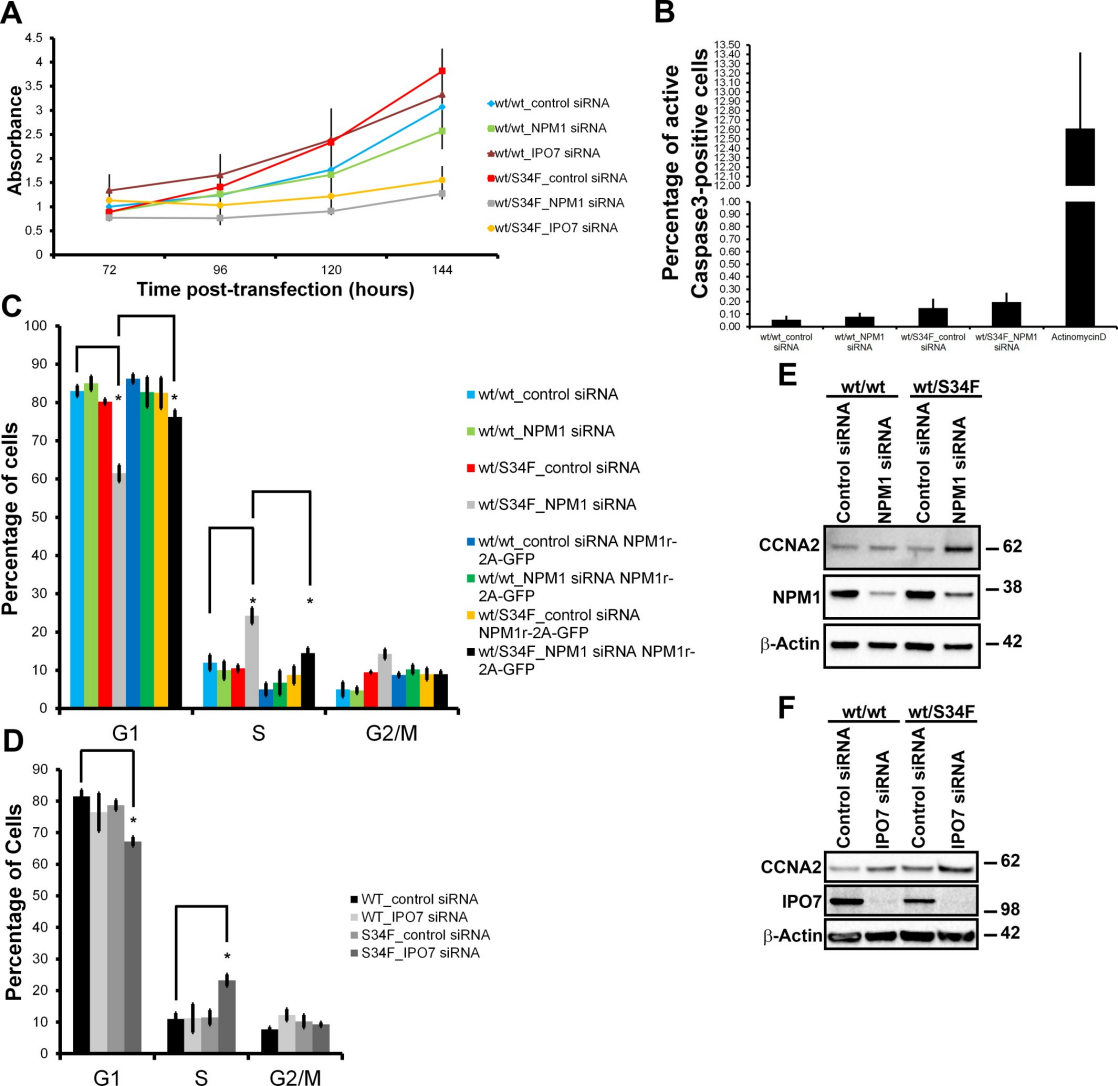
NPM1 or IPO7 silencing slows the proliferation of wt/S34F cells. (A) The viability of cells treated with siRNAs directed against NPM1 or IPO7 was assessed at the indicated time points post-siRNA transfection. Error bars represent the standard error of 3 independent experiments. (B) Quantification of the active Caspase-3 levels. Each bar represents the average and standard error of 3 independent experiments. (C) Cells that express either NPM1^r^-2A-GFP or empty vector were treated with siRNA directed against NPM1. 144 hours post-siRNA transfection, cells were pulsed with EdU for 10 minutes, and EdU incorporation was quantified. The fraction of cells in each phase of the cell cycle was calculated. Each bar represents the average and standard error of 4 independent experiments. *p < 0.05 using a paired 2-sided *t* test. (D) Cells were treated with siRNA directed against IPO7. 144 hours post-siRNA transfection, cells were pulsed with EdU for 10 minutes, and EdU incorporation was quantified. The fraction of cells in each phase of the cell cycle was calculated. Each bar represents the average and standard error of 4 independent experiments. *p < 0.05 using a paired 2-sided *t* test. (E) Cells treated with siRNA directed against NPM1 were lysed, and proteins were separated on SDS-PAGE and analyzed by immunoblot using antibodies against CCNA2, NPM1, and β-actin. (F) Cells treated with siRNA directed against IPO7 were lysed, and proteins were separated on SDS-PAGE and analyzed by immunoblot using antibodies against CCNA2, IPO7, and β-actin. The underlying data are in S1 Data. CCNA2, Cyclin A2; EdU, 5-ethynyl-2ʹ-deoxyuridine; GFP, green fluorescent protein; IPO7, Importin 7; NPM1, Nucleophosmin 1; siRNA, small interfering RNA; S34F, serine-34 to phenylalanine substitution; wt, wild-type.

The viability defect observed in wt/S34F cells lacking NPM1 can be explained by either an activation of apoptotic pathways or a defect in cell cycle progression. To distinguish between these possibilities, we first examined cellular viability over the time course of the knock-down experiment. First, wt/wt cells transfected with control siRNA had comparable growth with wt/wt cells depleted of either NPM1 or IPO7. In contrast, wt/S34F cells depleted of NPM1 or IPO7 had a drastically lower viability than wt/S34F cells treated with control siRNA (Fig 4A), consistent with our earlier observations (Fig 2D). Importantly, we observed a monotonic increase in the metabolic activity of the wt/S34F cells depleted of NPM1 or IPO7 over the time period of the measurement using the WST-1 assay. These findings are more consistent with slowed growth rates and not an induction of apoptotic pathways. We further confirmed the lack of apoptosis by comparing cleaved Caspase-3 levels. We chose Caspase-3 because it is one of the late apoptotic factors and thus is activated by both the extrinsic and intrinsic apoptotic pathways. We did not observe a difference in cleaved Caspase-3 levels between wt/wt and wt/S34F cells treated with either control or NPM1 siRNA, suggesting that apoptosis is not the cause of decreased viability in the wt/S34F cells lacking NPM1 activity (Fig 4B and S6A Fig). In contrast, actinomycin-D treatment (5 μg/mL) caused a robust up-regulation in cleaved Caspase-3 levels.

Next, we examined whether wt/S34F cells depleted of NPM1 had cell cycle progression defects. Cells were treated with 5-ethynyl-2ʹ-deoxyuridine (EdU) for 10 minutes to label the S-phase population [38]. The G1 and G2 cell populations were identified by quantifying the DNA fluorescence intensity using Hoechst (Fig 4C and S6B Fig). The depletion of NPM1 in wt/wt cells did not have a measurable effect on cell cycle progression. In contrast, the absence of NPM1 from the wt/S34F cells led to an increase in the fraction of S-phase cells and a concomitant decrease in the G1 population (Fig 4C). These results indicate that wt/S34F cells depleted of NPM1 take a longer time to complete the S phase. Expression of NPM1^r^-2A-GFP in wt/S34F when NPM1 is silenced restored the S-phase population to control siRNA levels (Fig 4C). We also observed elevated levels of the S-phase population in wt/S34F cells when IPO7 was silenced (Fig 4D and S6C Fig). We further confirmed the accumulation of cells in the S phase by examining the levels of Cyclin A2 (CCNA2), which is up-regulated in the S, G2, and early M phases. Indeed, CCNA2 levels were specifically up-regulated in wt/S34F cells depleted of NPM1 (Fig 4E) or IPO7 (Fig 4F). Silencing NPM1 or IPO7 expression in wt/wt cells did not have a detectable effect on CCNA2 levels. In summary, the absence of NPM1 or IPO7 caused the accumulation of wt/S34F cells in the S phase, leading to slower growth and a proliferative disadvantage.

**Fig 5:**
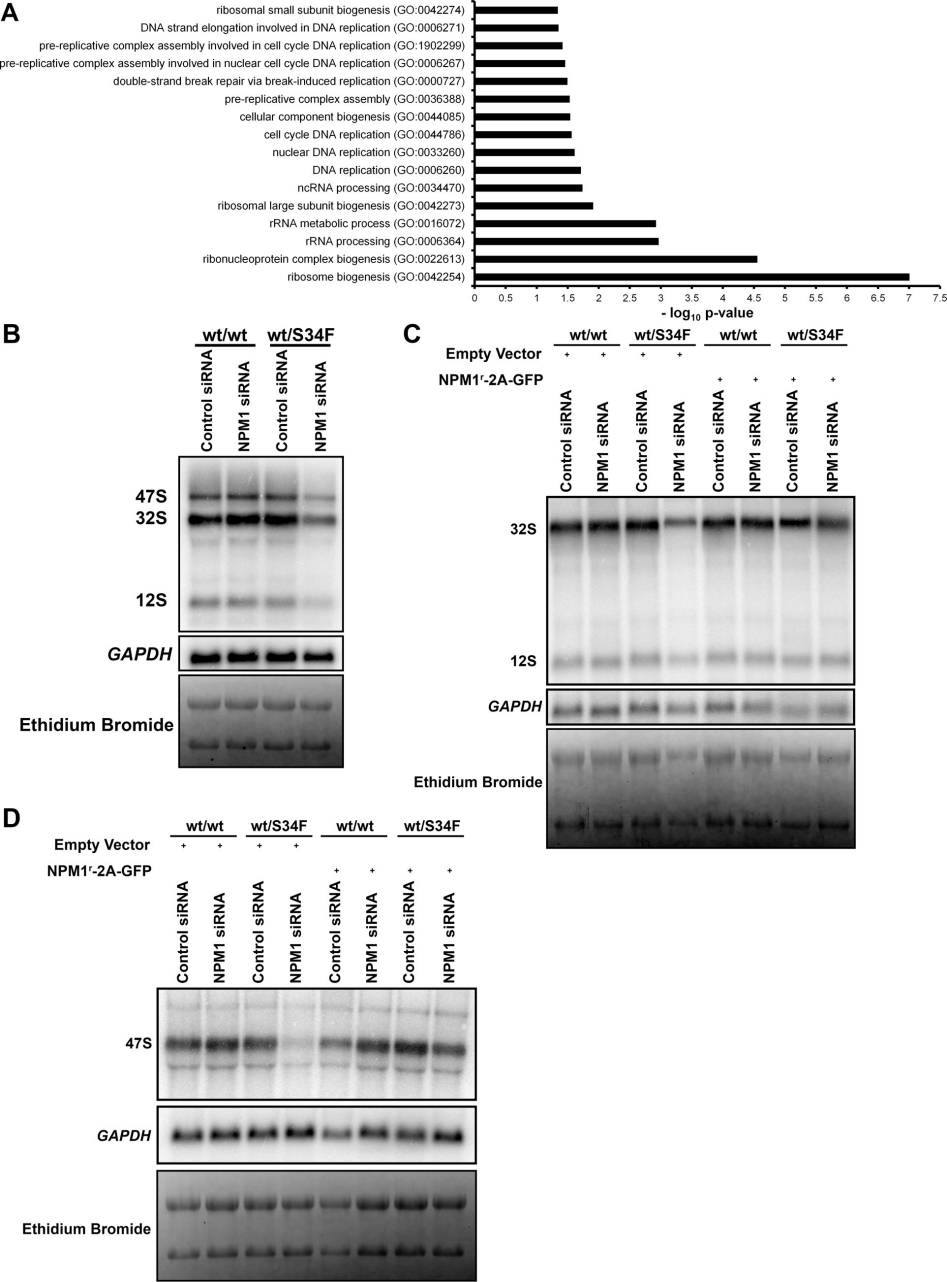
Silencing NPM1 impairs rRNA precursor levels in wt/S34F cells. (A) Statistically enriched GO terms using an FDR *p*-value < 0.05 threshold for down-regulated proteins in wt/S34F cells depleted of NPM1 in comparison to wt/wt cells. (B) The levels of the ITS2-containing rRNA precursors are decreased in wt/S34F cells depleted of NPM1. C) Overexpression of the RNAi-resistant NPM1^r^-2A-GFP restores the levels of the ITS2-containing rRNA precursors. (D) NPM1 silencing in wt/S34F decreases the level of the 47S rRNA and is rescued upon expression of the RNAi-resistant NPM1^r^-2A-GFP construct. The underlying data are in S1 Data. FDR, false discovery rate; *GAPDH*, glyceraldehyde-3-phosphate dehydrogenase; GFP, green fluorescent protein; GO, Gene Ontology; ITS2, internal transcribed spacer 2; NPM1, Nucleophosmin 1; RNAi, RNA interference; rRNA, ribosomal RNA; siRNA, small interfering RNA; S34F, serine-34 to phenylalanine substitution; wt, wild-type.

In order to dissect the molecular pathways that are perturbed in wt/S34F cells depleted of NPM1, we repeated our quantitative MS assays in wt/wt and wt/S34F cells after NPM1 knock-down. Of a total of 4,380 proteins detected in 3 replicates across 2 samples (6 total measurements), NPM1 depletion in wt/wt cells had a minimal effect on the cellular proteome. Only 7 and 13 proteins were up- and down-regulated, respectively, above an FC of 1.5. Importantly, these factors were not significantly enriched in any GO categories. In contrast, NPM1 silencing in wt/S34F caused 26 and 152 proteins to be up- and down-regulated, respectively, with an FC greater than 1.5 (S3 Table). A GO term analysis at an FDR *p*-value < 0.05 indicated that the down-regulated factors were statistically enriched for 2 major GO categories: “DNA replication coupled to cell cycle” and “ribosome biogenesis” (Fig 5A). The down-regulation of factors that couple DNA replication to cell cycle progression corroborates the accumulation of wt/S34F cells depleted of NPM1 in the S phase (Fig 4C and 4D). Furthermore, the negative enrichment for ribosome biogenesis factors suggested a direct mechanistic link to NPM1 function and the translational misregulation that arises from the S34F mutation, spurring us to investigate this observation in greater detail.

### Processing of the 28S rRNA precursors is impaired in wt/S34F cells depleted of NPM1

Ribosome biogenesis is initiated when RNA polymerase I transcribes ribosomal DNA (rDNA) into the 47S preribosomal RNA (rRNA) transcript that encompasses the 18S, 5.8S, and 28S flanked by 2 external (5ʹ external transcribed spacer [5ʹ ETS] and 3ʹ ETS) and interspersed by 2 internal spacers (internal transcribed spacer 1 [ITS1] and ITS2). The 18S rRNA and small subunit ribosomal proteins constitute the pre-40S ribosomal subunit. The 28S, 5.8S, and 5S (produced by RNA polymerase III), along with the large subunit ribosomal proteins, constitute the pre-60S subunit. NPM1 has been previously shown to bind the 28S rRNA [39] and regulate the processing of the 28S rRNA by promoting the localization of the ITS2-cleaving endoribonuclease Las1 like ribosome biogenesis factor (LAS1L) to the nucleolus [40]. Therefore, we reasoned that silencing NPM1 might cause a specific impairment in the processing of the 28S rRNA precursors.

We examined the levels of the 28S rRNA precursors by employing northern blot using a probe complementary in sequence to the ITS2 [41]. Silencing NPM1 in wt/wt cells did not cause a change in the levels of the ITS2-containing precursors, in agreement with previous studies in which NPM1 siRNA caused very modest changes in the 47S and 32S rRNA [14,16]. Importantly, these modest increases were not consistent for all NPM1-targeting siRNA sequences used in this study, suggesting a minimal effect for NPM1 on rRNA processing in cells that express wt U2AF1 [16]. In contrast, the levels of the ITS2-containing rRNA precursors in wt/S34F cells decreased after NPM1 was depleted (Fig 5B). The levels of glyceraldehyde-3-phosphate dehydrogenase *GAPDH* mRNA demonstrate equal loading across samples. However, the decrease in the ITS2 precursors did not cause a noticeable change in mature 28S and 18S rRNA levels, likely because of their long half-life ranging from 3 to 7 days [42,43]. Furthermore, the decrease in ITS2 levels in wt/S34F cells in the absence of NPM1 is rescued by expressing the RNAi-resistant NPM1^r^-2A-GFP (Fig 5C). We also examined the levels of the primary 47S rRNA transcript using a probe that is complementary to the 5ʹ ETS [44]. Silencing NPM1 in wt/S34F cells caused a marked decrease in the 47S rRNA (Fig 5D). In contrast, 47S rRNA was comparable between wt/wt cells, wt/S34F cells, and wt/wt cells depleted of NPM1. Quantification of band intensities is shown (S7 Fig). These results indicate that the wt/S34F cells are dependent on NPM1 for proper ribosome biogenesis, as shown from the quantitative MS (Fig 5A) and the rRNA precursor levels (Fig 5B, 5C and 5D).

### The U2AF1-S34F mutation up-regulates mRNA translation in mouse myeloid progenitors and is mutually exclusive with NPM1 mutations in human patients

Our previous studies were carried out in HBECs. These cells have the benefit of being immortalized non-transformed human cell lines, which might better phenocopy the context in which initiating mutations such as U2AF1-S34F occur. However, U2AF1 mutations (like all splicing factor mutations) occur most frequently in the context of myeloid malignancy, so we examined the functional relevance of these findings with 2 additional approaches: experimental perturbation of immortalized mouse myeloid progenitor cells in tissue culture and analysis of human patient somatic mutation data.

**Fig 6:**
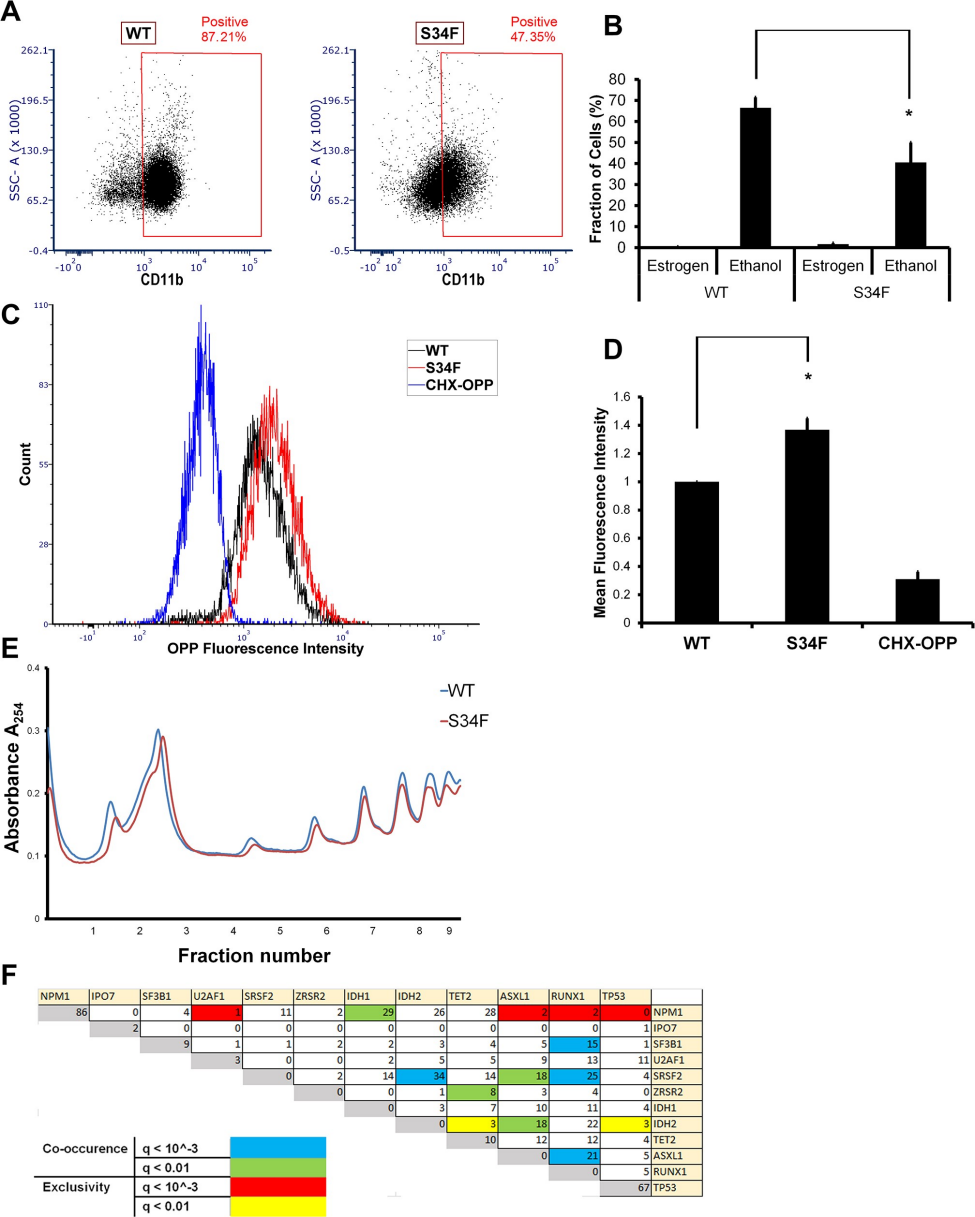
The U2AF1-S34F mutation up-regulates mRNA translation in immortalized myeloid cells. (A) The U2AF1-S34F mutation impairs the ability of myeloid progenitor cells to differentiate. (B) Quantification of A. Each bar represents the average and standard error of at least 4 independent experiments. *p < 0.05 using a paired 2-sided *t* test. The underlying data are in S1 Data. (C) The U2AF1-S34F mutation up-regulates mRNA translation. (D) Quantification of C. Each bar represents the average and standard error of 4 independent experiments. *p < 0.05 using a paired 2-sided *t* test. The underlying data are in S1 Data. (E) Absorbance at 254 nm of sucrose density gradient fractions from wt and S34F myeloid cells. The underlying data are in S1 Data. (F) Correlation matrix showing co-occurring and mutually exclusive mutations of the indicated factors. The underlying data are in S4 Table. U2AF1 and NPM1 mutations are mutually exclusive in MDS and AML patients (q-value < 0.001). AML, Acute Myeloid Leukemia; ASXL1, Additional sex combs like transcriptional regulator 1; CD, cluster of differentiation; CHX, cycloheximide; IDH, isocitrate dehydrogenase; IPO7, Importin 7; MDS, Myelodysplastic Syndrome; NPM1, Nucleophosmin 1; OPP, O-propargyl-puromycin; RUNX1, Runt-related transcription factor 1; SF3B1, splicing factor 3 subunit 1; SRSF2, serine and arginine rich splicing factor 2; SSC-A, side scatter-Area; S34F, serine-34 to phenylalanine substitution; TET2, Tet methylcytosine dioxygenase 2; TP53, tumor protein 53; U2AF1, U2 Small Nuclear RNA Auxiliary Factor 1; wt, wild-type; ZRSR2, zinc finger CCCH-type, RNA binding motif and serine/arginine rich 2.

First, we sought to examine whether the U2AF1-S34F mutation up-regulates mRNA translation in myeloid cells. We employed Homeobox B8 (Hoxb8) overexpression to immortalize myeloid progenitor cells as previously described [45]. These myeloid progenitors were derived from bone marrow of transgenic mice that express the human cDNA of either U2AF1-wt or U2AF1-S34F from the mouse collagen, type I, alpha 1 (*Col1a1*) locus [46]. Hoxb8 is expressed as a fusion to the estrogen-binding domain of the estrogen receptor (ER). In the presence of estrogen, Hoxb8 arrests myeloid differentiation and maintains the cells at the granulocyte/macrophage progenitor (GMP) stage. Upon estrogen withdrawal, the cells differentiate into neutrophils and macrophages that express the panmyeloid marker CD11b [45]. Strikingly, we observed that the U2AF1-S34F mutation impairs myeloid differentiation and maintains the mutant cells in a less differentiated state. In the absence of estrogen, i.e., under differentiation conditions, about 66% of wt cells expressed the panmyeloid differentiation marker CD11b. In contrast, only 47% of S34F cells expressed CD11b (Fig 6A and 6B). Thus, S34F GMP cells are not able to differentiate to the same extent as wt cells, indicating that the S34F missense mutation is sufficient to recapitulate aspects of the MDS cytopenia phenotype in vitro.

Next, we examined nascent polypeptide biosynthesis in wt and S34F myeloid progenitor cells. We treated the myeloid progenitors with the puromycin analog, O-propargyl-puromycin (OPP), which incorporates into nascent polypeptide chains [8]. The OPP-containing polypeptides are then chemically reacted to a fluorophore and detected by flow cytometry as previously described [8]. This assay is functionally similar to the AHA assay used previously but works better for suspension cells using flow cytometry. Similar to HBECs, S34F myeloid progenitors had higher mRNA translation levels in comparison to wt cells (Fig 6C and 6D). However, polysome profiles were unchanged between the cell lines (Fig 6E). Cells pretreated with 100 μg/mL cycloheximide (CHX) for 1 hour before adding OPP had a lower signal, confirming that the OPP signal corresponds to nascent polypeptides. Again, the elevated mRNA translation levels in S34F cells shows that the U2AF1-S34F mutation in progenitor cells in vitro is sufficient to recapitulate the elevated levels of polypeptide production observed in hematopoietic stem cells derived from MDS patients [10].

Second, because the S34F cells require NPM1 for their viability, we hypothesized that the S34F mutation should not co-occur in cancer patients with NPM1 loss of function mutations. Both *U2AF1* and *NPM1* are frequently mutated in patients with malignancies of the myeloid lineage. According to the cBioPortal database [47,48] reporting of 4 studies of adult MDS and AML patients [4,49–51], *U2AF1* mutations occur in 5% and *NPM1* is mutated in 22% of all myeloid malignancy patients. Despite the high frequency of these mutations, we found that *U2AF1* and *NPM1* mutations co-occurred at a probability lower than that expected by chance (Fig 6F), likely because of the deleterious viability defect caused by abolishing NPM1 function in cells harboring U2AF1 mutations. Statistical significance was based on q-values computed by the cBioPortal database. Raw values from the cBioPortal database are shown (S4 Table). This analysis recapitulated other previously observed mutual dependencies. For example, serine and arginine rich splicing factor 2 (*SRSF2*) and isocitrate dehydrogenase 2 (*IDH2*) are more likely to co-occur [52]. Notably, *NPM1* mutations were mutually exclusive with *U2AF1* mutations, but not with other commonly mutated splicing factors such as splicing factor 3 subunit 1 (*SF3B1*), *SRSF2*, and zinc finger CCCH-type, RNA binding motif, and serine/arginine rich 2 (*ZRSR2*). Taken together, these analyses indicate that both the role of U2AF1-S34F in translation regulation and the interaction between U2AF1 and NPM1 are relevant in a disease context.

## Discussion

Although U2AF1 mutations have been implicated as driver mutations in lung cancer and MDS, the role of these mutations in disease progression has remained elusive. Here, we demonstrate an unanticipated functional connection between U2AF1, translation initiation, and ribosome biogenesis. We show that the U2AF1-S34F mutation leads to a substantial change in the translation machinery, resulting in changes in mRNA translation at the single-cell level in both HBECs and mouse myeloid progenitor cells, thus defining a unique molecular phenotype for this splicing factor mutation. Furthermore, wt/S34F cells require ribosome biogenesis factors such as NPM1 and IPO7 to proliferate. A quantitative proteomic analysis demonstrates that silencing NPM1 in wt/S34F cells causes a statistically significant decrease in factors that regulate ribosome biogenesis and cell cycle progression, and this observation is corroborated by concomitant defects in rRNA processing. This in vitro “nononcogene addiction” of the S34F mutation to NPM1 is validated by human patient data demonstrating that *U2AF1* and *NPM1* mutations are mutually exclusive in myeloid malignancies.

To our knowledge, this U2AF1-S34F mutation is the first missense mutation to recapitulate a translation phenotype in myeloid malignancy. Although the translation machinery has long been known to be misregulated in cancer [53] and is the molecular basis for the inherited ribosomeopathies that affect myeloid differentiation, these latter genetic alterations are typically germ-line deletions of ribosomal subunits. Somatic mutations in the translation machinery or related translation initiation factors have not been reported in MDS or AML. Importantly, we show this phenotype in the context of immortalized mouse myeloid progenitors in which the S34F mutation is able to phenocopy MDS-linked symptoms such as altered differentiation leading to cytopenias, anemias, and neutropenias. However, the molecular changes in the abundance of translation initiation proteins induced by the S34F mutation are not easily interpreted. For example, this family of proteins is decreased in comparison to wt/wt cells, but not evenly so. A number of eukaryotic initiation factors, helicases, and NPM1 are elevated. A subset of ribosomal proteins is down-regulated, whereas other ribosomal proteins do not change. Yet, the net output is increased peptide biosynthesis as measured by the incorporation of the amino acid analog (AHA) or tRNA analog (OPP). Likewise, a puromycin incorporation assay indicates both increases in peptide biosynthesis and a shift in mobility consistent with smaller peptides, indicating that elevated translation is concomitant with changes to the proteome. The change to translational activity is evocative of numerous studies showing that translational regulation is particularly important during hematopoiesis [8–10,54]. The nature of this increased translation that we and others observe is still unclear. Are certain functional categories up-regulated? Does the increased translation result in full-length or truncated proteins? Additional work is necessary to precisely answer these questions.

Previous work has identified p53 as an important mediator of ribosome dysfunction, especially in bone marrow failure [34,55]. For example, p53 accumulates in the erythroid lineage upon knock-down of *RPS14*, the ribosomal protein gene deleted in a subtype of MDS called 5q-syndrome [34]. In addition, erythroid differentiation defects could be rescued by pharmacological inhibition of p53. However, whereas NPM1 has been reported to regulate p53 activity either directly [56] or through ARF [19–21], several lines of evidence argue against a role for p53 in the viability defect of wt/S34F cells depleted of NPM1. First, abrogating NPM1 expression causes comparable levels of p53 induction in both the wt/wt and wt/S34F cells (Fig 3A). Second, co-depletion of NPM1 and p53 did not rescue the viability defect observed in single NPM1 depletion (Fig 3B and 3C). Third, p53 activation by 20 μM Nutlin3A caused a comparable impairment of the viability of wt/wt and wt/S34F cells (Fig 3D, 3E and 3F). Thus, we conclude that the phenotype we observe, namely the dependence of the S34F mutation on functional NPM1, is p53-independent.

Why are ribosome biogenesis factors specifically required for the viability and proliferation of wt/S34F cells, but not wt/wt U2AF1 cells? We note that these experiments were performed in bronchial epithelial cells, which are an immortalized but nontransformed cell line [22]. This system might better recapitulate splicing factor mutations, which usually occur early in disease progression [57]. In tissues, cells must strike a balance between proliferation, differentiation, and self-renewal, for example, as demonstrated in hematopoiesis [9]. This balance is altered in MDS, and hematopoietic stem cells derived from MDS patients have elevated mRNA translation levels [10]. Our isogenic cell culture system recapitulates this finding with only a single nucleotide change in the *U2AF1* gene. Moreover, we speculate that ribosome biogenesis factors such as NPM1 or IPO7, which are not required for cell viability under basal growth levels [15,32], become crucial when mRNA translation levels are elevated due to the U2AF1-S34F mutation. The absence of NPM1 impairs rRNA synthesis or processing in wt/S34F cells, and this slows cell proliferation and causes their accumulation in the S phase. Indeed, NPM1 has been shown to localize to centrosomes and regulate their duplication, suggesting potential links between ribosome biogenesis and cell division factors [15,58]. Moreover, mTOR, another growth-promoting signaling pathway, has been shown to up-regulate NPM1 expression [59]. It should be also noted that abrogating the expression of the small ribosome subunit protein RPS6 impairs cell division in mice [60].

Our results suggest a model in which the U2AF1-S34F mutation alters the translation of mRNAs that code for many translation and ribosome biogenesis factors, leading to an increase in protein synthesis (Fig 1). Although NPM1 silencing was not sufficient to perturb cell viability [14,15] or rRNA processing [14,16] in various cell lines that express wt U2AF1, NPM1 silencing has been shown to alter nucleolar shape [61] and the localization of ribosome biogenesis factors to the nucleolus [40]. We propose increased cellular dependence on NPM1 under conditions of elevated mRNA translation such as in the presence of U2AF1-S34F. NPM1 silencing in wt/S34F cells impairs rRNA synthesis or processing (Fig 5B, 5C and 5D), and ribosome biogenesis factors are down-regulated (Fig 5A) potentially at the transcriptional, translational or post-translational levels in wt/S34F cells. Furthermore, the down-regulation of factors that couple DNA replication to cell cycle progression causes the accumulation of wt/S34F cells depleted of NPM1 in the S phase (Fig 4) and their decreased viability (Fig 2). The dependence of wt/S34F cells on NPM1 accounts for the mutual exclusivity of U2AF1 and NPM1 mutations in MDS and AML patients (Fig 6F). In sum, this study uncovered a novel, to our knowledge, dependence of the cancer-associated U2AF1 mutations on ribosome biogenesis to maintain a highly proliferative state.

## Materials and methods

### Plasmids and cells lines

The plasmid expressing the NPM1^r^-2A-GFP reporter was generated by fusing the cDNA of NPM1 amplified from HBEC cDNA library upstream of a T2A-GFP synthetic construct (IDT, Coralville, IA, USA) using Superscript III reverse transcriptase according to the manufacturer’s instructions (Thermo Scientific, Waltham, MA, USA). RNAi resistance was conferred by introducing silent mutations in the siRNA-targeting sequence. This construct was subsequently inserted into a lentiviral transfer plasmid that has the ubiquitin C promoter. The NPM1 luciferase reporter was generated by fusing 2 synthetic constructs (IDT) into the ubiquitin C lentiviral transfer plasmid. The first construct was composed of human NPM1 exon 1 sequence fused to *Renilla* luciferase. The second construct was composed of CrPV IRES fused to firefly luciferase [30]. The lentiviral plasmids were packaged into lentiviral particles using HEK293T cells. Virus titer was measured, and the viruses were transduced into HBECs using 8 μg/mL polybrene. HBEC lines were cultured in keratinocyte serum-free medium supplemented with bovine pituitary extract and epidermal growth factor (Gibco, Gaithersburg, MD, USA). Wt/wt, wt/S34F and wt/S34F- HBEC cells were previously reported [3]. For translation inhibition experiments, cells were incubated with puromycin (100 μg/mL) for 30 minutes prior to AHA treatment. Nutlin3A treatment was for 36 hours at the indicated doses. For Caspase-3 experiments, cells were treated with actinomcycin D (5 μg/mL) for 12 hours. For siRNA experiments, cells were treated with 25 pmol smartpool siRNA (Dharmacon, Lafayette, CO, USA) and Lipofectamine RNAiMax transfection reagent (Thermo Scientific). Knock-down efficiency was assessed after 6 days by western blotting. The following siRNA pools were used: NPM1 (M-015737-01-0005), IPO7 (L-012255-00-0005), TP53 (L-003329-00-0005), and RBM10 (E-009065-00-0005) (Dharmacon). Hoxb8-immortalized myeloid cells were generated as previously described [45]. Briefly, bone marrow mononuclear cells were harvested from murine models [46] and stimulated in RPMI supplemented with 10 ng/mL IL6, IL3, murine stem cell factor (mSCF), 10% fetal bovine serum (FBS), and 1% penicillin/streptomycin for 36 hours. Cells were then transduced in a 12-well plate coated with fibronectin (250,000 cells in 500 μL opti-MEM) with 1 mL of HoxB8-ER retrovirus and 8 μg/mL polybrene by spinfection. After spinfection, 3 mL of RPMI supplemented with 10% FBS, 1% pen/strep, 50 ng/mL mSCF, and 0.5 μM beta-estradiol was added. The cells were cultured in RPMI 1640 supplemented with 10% FBS, 1% mSCF, and 1 μM β-estradiol (Sigma-Aldrich, St. Louis, MO, USA). The cells were grown in 1 μg/mL doxycycline for 24 hours to induce the U2AF1 transgene expression. Cells were induced to differentiate by culturing in the absence of β-estradiol for 6 days before cells were stained for CD11b (BioLegend, San Diego, CA, USA) and analyzed on the LSRII flow cytometer (BD Biosciences, San Jose, CA, USA). Data analysis was performed using FCS Express (De Novo Software).

### Polysome profiling

Polysomes were frozen using 100 μg/mL CHX for 10 minutes. Cells were harvested and lysed in 20 mM Tris, 130 mM KCl, 0.5% NP40, 10% deoxycholate, 100 μg/mL CHX, and 10 μL/mL RNasin (Promega, Madison, WI, USA). Lysate concentration was measured and equal OD260 units of wt/wt and wt/S34F were loaded onto 10%–50% sucrose prepared using a BioComp Gradient Master (BioComp, Fredericton, NB, Canada). Gradients were spun at 40,000 RPM (Beckman Coulter, Brea, CA, USA), fractionated and the absorbance at 254 nm measured using a gradient fractionator (BioComp).

### Polysome profile analysis

The following steps are used to compute changes in translation efficiency from polysome sequencing profiles [6]:

1. For each sample (wt/wt, wt/s34f, wt/s34f-), 12 fractions were collected from the sucrose gradient, and the bottom/heaviest 10 fractions were sequenced.
2. Fractions 10–12 were pooled to increase coverage, resulting in 8 fractions in the final analysis. Fraction 5 and 6 correspond to the monosome fractions.
3. Reads mapping to particular message are normalized by the total reads in that sample and multiplied by 1 × 10^6^, generating counts per million (CPM) for each transcript in each fraction.
4. The CPM in each fraction constitutes the polysome profile (poly[x], where “x” designates the sucrose fraction), which is now normalized for read depth across fractions/samples.
5. This profile can be further normalized by a weighting function that accounts for the nonlinear relationship between sedimentation coefficient, position in the sucrose gradient, and molecular mass: S is approximately x^3/2^, where *S* is the sedimentation coefficient in Svedrup [62]. Here, we use the following weights for each fraction after pooling: *w* = [0.6, 1.0, 1.6, 2.2, 2.9, 3.7, 4.5, 19.0].
6. The final numeric value, reflecting translation efficiency, is ∑w_i_poly(x_i_). This number, generated for each transcript in each of the 3 cell lines, is the basis for clustering in Fig 1A. Hierarchial clustering was done using ClustVis [63].
7. Alternatively, a simpler measure of translational efficiency is reported (S1 Fig), consisting only of the polysome/monosome ratio, i.e., without a weighted estimator. The monosome fractions are taken as w_2_ and w_3_, and the polysome fractions are w_5_, w_6_, w_7_, and w_8_.

Average polysome profiles were generated from quintiles consisting of 35 genes. Error bars are generated with bootstrapping over 30 random replicates.

### GSEA

GSEA analysis was done with GSEA v. 4.0.3 from The Broad Institute using the “GSEA on a pre-ranked gene list” option [27]. Gene set database is c5.bp.v7.0.symbols.gmt run with 1,000 perturbations. The enrichment statistic is “weighted.” Accepted gene sets contained between 15 and 500 members.

### Luciferase assay

Luciferase plasmids were introduced by lentiviral transduction. Equal number of cells were plated in a 96-well dish. After 24 hours, cells were treated with the Dual-Luciferase Reporter Assay System as per manufacturer’s instructions (Promega). Luciferase activity was measured by Victor plate reader (Perkin Elmer, Waltham, MA, USA). To quantify *luciferase* mRNA levels, RNA was isolated by Trizol (Thermo Scientific), treated with DNaseI and reverse transcribed using Superscript III (Thermo Scientific) according to the manufacturer’s instructions using random hexamers. qPCR was done using iTaq SYBR Green master mix (Bio-Rad, Hercules, CA, USA) and run on a CFX96 PCR detection system (Bio-Rad). *Luciferase* mRNA levels were normalized to tubulin mRNA. Primer sequences for luciferase are TAACGCGGCCTCTTCTTATTT and GATTTGCCTGATTTGCCCATAC. Primer sequences for *Tubulin* mRNA are CGATATTGAGCGTCCAACCTAT and TTCAGGGCTCCATCAAATCTC.

### AHA metabolic labeling

Protein biosynthesis was assessed by AHA pulse labeling as previously described [28] with the following modifications. Cells were plated on coverslips (VWR, Radnor, PA, USA). After 24 hours, cells were pulsed with AHA for 1 hour. Cells were then fixed in 4% paraformaldehyde in PBS, permeabilized in 0.1% TritonX in PBS, and blocked in blocking reagent that contains 2% BSA and 5% donkey serum in PBS for 3 hours. Cells were washed once in PBS. The click chemistry reaction was composed of Tris(2-carboxyethyl)phosphine (TCEP) (Thermo Scientific) and Tris[(1-benzyl-1H-1,2,3-triazol-4-yl)methyl]amine (TBTA) (Sigma-Aldrich). The reaction was allowed to proceed for 1 hour. Coverslips were washed in 0.1% TritonX in PBS 3 times, 15 minutes each and mounted on microscopy slides using DAPI-containing mounting solution.

### Quantitative MS

Quantitative MS was done according to the NCI MS core standard protocol. Briefly, cells were lysed, and total protein was quantified using bicinchoninic acid (BCA) reagent (Thermo Scientific). The samples were reduced using DTT (Thermo Scientific), alkylated with iodoacetamide (Thermo Fisher Scientific), and trypsinized (Promega) overnight. TMT tags were conjugated to the peptides and cleaned up using peptide desalting columns (Thermo Scientific). The first dimensional separation of the peptides was performed using a Waters Acquity UPLC system coupled with a fluorescence detector (Waters, Milford, MA, USA). The dried peptide fractions were reconstituted in 0.1% TFA and subjected to nanoflow liquid chromatography (Thermo Easy nLC 1000; Thermo Scientific) coupled to high-resolution tandem MS (Q Exactive, HF; Thermo Scientific). Peptides were separated using a second-dimension low pH gradient using a 2%–40% ACN over 120 minutes in mobile phase, containing 0.1% formic acid at 300 nl/min flow rate. MS scans were performed in the Orbitrap analyzer at a resolution of 120,000 with an ion accumulation target set at 3 × 10^6^ and max IT set at 50 ms over a mass range of 200–1,800 m/z, followed by MS/MS analysis at a resolution of 45,000 with an ion accumulation target set at 1 × 10^5^, max IT set at 120 ms and first fixed mass set at 105 m/z. MS2 precursor isolation width was setup at 0.7 m/z, normalized collision energy was 29, and charge state 1 and unassigned charge states were excluded.

### MS data analysis

Acquired MS/MS spectra were searched against a human uniprot protein database along with a contaminant protein database, using an SEQUEST and percolator validator algorithms in the Proteome Discoverer 2.2 software (Thermo Scientific,). The precursor ion tolerance was set at 10 ppm, and the fragment ions tolerance was set at 0.02 Da along with methionine oxidation included as dynamic modification and TMT6 plex (229.163 Da) set as a static modification of lysine and the N-termini of the peptide.

### Image acquisition and analysis

Slides were imaged using a custom epifluorescence microscope equipped with a Plan-Apochromatic 40× (NA 1.4) objective (Carl Zeiss, Oberkochen, Germany), illuminated with a LED light source (Model Spectra-6LCR-SA; Lumencor, Beaverton, OR, USA) and the emitted fluorescence collected with a CMOS camera (Hamamatsu ORCA-Flash 4.0; Hamamatsu, Hamamatsu City, Japan). The microscope was controlled using μManager [64]. A custom pipeline was employed to compute total fluorescence intensity per cell using Cellprofiler (Broad Institute) [65].

### Western blotting

Cells were lysed in 1× RIPA buffer, protein lysate quantified using the BCA kit (Thermo Scientific), boiled in SDS, and run on SDS-PAGE. Proteins were transferred onto nitrocellulose membrane, blocked in 5% milk, and probed with the following antibodies: NPM1 (1:1,000; Abcam, Cambridge, UK), IPO7 (1:1,000; Abcam), p53 (1:500; Bethyl Laboratories, Montgomery, TX, USA), CCNA2 (1:1,000; BD Biosciences), RBM10 (1:1,000; Sigma-Aldrich), puromycin (1:1,000; Sigma-Aldrich), tubulin (1:1,000; Abcam), and actin (1:10,000; Sigma-Aldrich). For SUnSET, cells were treated with 10 μg/mL puromycin for 1 hour before lysis. The CHX-puromycin sample was treated first with 100 μg/mL CHX for 1 hour. Subsequently, fresh medium containing 10 μg/mL puromycin and 100 μg/mL CHX was added to the cells for another 1 hour before cells were lysed.

### Viability assay

To assess changes in cell viability, cells were counted using an automated cell counter (Bio-Rad), and an equal number of cells were plated in a 96-well plate. On the sixth day of the siRNA experiment, fresh medium was added that contained 1:10 dilution of the WST-1 reagent (Sigma-Aldrich). Cells were incubated for different time points before the absorbance was measured at 450 nm using a plate reader (Bio-Rad).

### Apoptosis assay

Apoptotic induction was examined by checking for cleaved Caspase-3 levels using a PE-conjugated anti-Caspase-3 antibody per the manufacturer’s instructions (BD Biosciences). PE levels were examined using an LSRII flow cytometer (BD Biosciences). Data analysis was performed using FCS Express (De Novo Software, Pasadena, CA, USA).

### Cell cycle analysis

Cell cycle progression was examined using the Click-it EdU Alexa Fluor 647 kit (Thermo Scientific). Briefly, cells were plated on cover slips. After 24 hours, cells were pulsed in EdU for 10 minutes and then fixed in 4% paraformaldehyde in PBS, permeabilized in 0.1% TritonX in PBS. Cells were then washed twice in 2% BSA in PBS. The EdU was reacted with azide-conjugated fluorophore in the presence of copper sulfate. The click chemistry reaction was allowed to proceed for 1 hour. Cells were washed twice in 2% BSA in PBS. DNA was labeled using Hoechst at a dilution of 1:10,000 mounted on Prolong Gold mounting solution (Thermo Scientific). The fraction of cells in each stage of the cell cycle was quantified using a previously published MATLAB (The MathWorks, Natick, MA, USA) analysis pipeline [66] and is available at https://github.com/scappell/Cell_tracking.

### GO term analysis

The genes that were down-regulated at an FC of 1.5 in wt/S34F cells depleted of NPM1 in comparison to wt/wt treated with control siRNA were uploaded into the Panther Database (www.geneontology.org) [67]. The Panther overrepresentation test was used with the test type = FISHER and the correction = FDR.

### Northern blotting

Equal numbers of cells were harvested for all conditions using the Luna Automated Cell Counter (Logos Biosystems). Total RNA was extracted using Trizol (Thermo Scientific) according to the manufacturer’s instructions. The RNA was separated on a denaturing gel and transferred to nylon membrane (GE Healthcare, Chicago, IL, USA) overnight as previously described [68]. The membrane was hybridized to a ^32^P-labeled probe overnight and imaged using a Typhoon phosphoimager (GE Healthcare). To probe for the ITS2, an oligonucleotide was 5ʹ-labeled using PNK enzyme (New England Biolabs, Ipswich, MA, USA). The ITS2 probe was previously reported [41], and its sequence is GAGGGAGGAACCCGGACCGCAGGCGGCGGCCACGGGAACTCGGCCCGAGCCGGCTCTCTC. The 5ʹ ETS probe sequence was previously reported [44], and its sequence is GGCGAGCGACCGGCAAGGCGGAGGTCGACCCACGCCACACGTCGCACGAACGCCTGTC. The pET30-2-GAPDH plasmid,a gift from David Sabatini (Addgene plasmid # 83910; Addgene, Watertown, MA, USA), was used to generate the GAPDH probe[69], and gene-body labeling was performed using the Prime-A-Gene kit (Promega). rRNA precursor band intensity was measured using FIJI [70] and normalized to *GAPDH* mRNA band intensity.

### OPP labeling

Immortalized myeloid cells were treated with 10 μM OPP (Click Chemistry Tools, Scottsdale, AZ, USA) for 1 hour. Cells were fixed and permeabilized. Click chemistry was employed to conjugate a fluorophore to the OPP-containing polypeptides according to the manufacturer’s instructions (Thermo Scientific). Cells were washed 5 times in PBS and analyzed on the LSRII flow cytometer (BD Biosciences). Data analysis was performed using FCS Express (De Novo Software). For CHX-OPP samples, cells were treated first with 100 μg/mL CHX for 1 hour. Subsequently, fresh medium containing 10 μM OPP and 100 μg/mL CHX for another 1 hour before cells were fixed.

### Mutual exclusivity of *U2AF1* and *NPM1* mutations

Cancer patient data were retrieved from cBioPortal [47,48] (www.cbioportal.org) as of October 4th, 2019. All adult AML and MDS patient studies were selected. These include “Acute myeloid leukemia or myelodysplastic syndromes (Wash U, 2016)” [49], “Acute myeloid leukemia (OHSU, 2018)” [50], “Acute myeloid leukemia (TCGA, PanCancer Atlas)” [51], and “Myelodysplasia (UTokyo, Nature 2011)” [4]. Statistical significance was based on q-values computed by the cBioPortal website. Raw values from the cBioPortal website are shown in S4 Table.

## Supporting information

S1 Fig Recalculation using a more conservative polysome estimator. (A) Heat map and hierarchical clustering of the polysome/monosome estimator applied to RNA-seq from polysome fractions. The genes are the 175 translation initiation genes present in all 8 fractions of each sample (wt/wt, wt/S34F, wt/S34F-) cells (24 total measurements). Rows are centered; no scaling is applied to rows. Rows are clustered using Euclidean distance and Ward linkage. Columns are clustered using correlation distance and average linkage. 175 rows, 3 columns. (B) Principal component analysis of polysome/monosome estimator applied to RNA-seq from polysome fractions. No scaling is applied to rows; SVD with imputation is used to calculate principal components. *X* and *Y* axis show PC 1 and PC 2, which explain 78.3% and 21.7% of the total variance, respectively. The underlying data are in S5 Table. PC, principal component; RNA-seq, RNA sequencing; SVD, singular value decomposition; S34F, serine-34 to phenylalanine substitution; wt, wild-type.
(TIF)

S2 Fig Polysome profiles for the top, middle, and bottom quintiles. (A) Polysome profile for the top quintile mRNAs. (B) Polysome profile for the middle quintile mRNAs. (C) Polysome profile for the bottom quintile mRNAs. The underlying data are in S1 Data.
(TIF)

S3 Fig Full results of the GSEA analysis on MS data. (A) The top 4 GO categories show similar FDR and FWER and contain many of the same genes. The underlying data are in S2 Table. (B) The overlap between the GO categories of “nuclear transcribed mRNA catabolic process nonsense mediated decay” and “translation initiation” is shown as a Venn diagram. The 17 genes that are present in “nuclear transcribed mRNA catabolic process nonsense mediated decay,” but not “translation initiation,” are listed. FDR, false discovery rate; FWER, family-wise error rate; GO, Gene Ontology; GSEA, gene set enrichment analysis; MS, mass spectrometry
(TIF)

S4 Fig Puromycin labeling of nascent polypeptides in wt/wt and wt/S34F cells. (A) Representative western blot of puromycin-labeled nascent polypeptides. (B) Quantification of the signal intensity. Each bar represents the average and standard error of 3 independent experiments. The underlying data are in S1 Data. CHX, cycloheximide; S34F, serine-34 to phenylalanine substitution; wt, wild-type.
(TIF)

S5 Fig RT-qPCR showing comparable *NPM1-luciferase* mRNA levels in wt/wt, wt/S34F, and wt/S34F- cells. The *NPM1-luciferase* mRNA levels were normalized to *TUBA1A* mRNA. Each bar represents the average and standard error of 3 independent experiments. The underlying data are in S1 Data. *NPM1*, Nucleophosmin 1; RT-PCR, reverse transcription–quantitative PCR; S34F, serine-34 to phenylalanine substitution; *TUBA1A*, tubulin alpha 1a; wt, wild-type.
(TIF)

S6 Fig Representative gating strategy used in Caspase-3 and EdU experiments. (A) Gating strategy from a representative cleaved Caspase-3 experiment. (B) Gating strategy from a representative EdU labeling experiment for NPM1 siRNA. (C) Gating strategy from a representative EdU labeling experiment for IPO7 siRNA. DMSO, dimethyl sulfoxide; EdU, 5-ethynyl-2ʹ-deoxyuridine; IPO7, Importin 7; NPM1, Nucleophosmin 1; PE-A, Phycoerythrin-Area; siRNA, small interfering RNA; SSC-A, side scatter-Area
(TIF)

S7 Fig Quantification of northern blot band intensity. (A) Quantification of the ITS2 northern blot showing wt/wt and wt/S34F cells treated with control or NPM1 siRNA. Each bar represents the average and standard error of 3 independent experiments. *p < 0.05 using a paired 2-sided *t* test. (B) Quantification of the ITS2 northern blot showing wt/wt and wt/S34F cells that overexpress an empty vector or the NPM1^r^-2A-GFP reporter and were treated with control or NPM1 siRNA. Each bar represents the average and standard error of 3 independent experiments. *p < 0.05 using a paired 2-sided *t* test. (C) Quantification of the 5ʹ ETS northern blot showing wt/wt and wt/S34F cells that overexpress an empty vector or the NPM1^r^-2A-GFP reporter and were treated with control or NPM1 siRNA. Each bar represents the average and standard error of 3 independent experiments. *p < 0.05 using a paired 2-sided *t* test. The underlying data are in S1 Data. A.U., auxiliary unit; EV, empty vector; GFP, green fluorescent protein; ITS2, internal transcribed spacer 2; NPM1, Nucleophosmin 1; siRNA, small interfering RNA; S34F, serine-34 to phenylalanine substitution; wt, wild-type; 5ʹ ETS, 5ʹ external transcribed spacer
(TIF)

S1 Table Quantification of translation efficiency from polysome profiling data using the weighted polysome estimator in wt/wt, wt/S34F, and wt/S34F- cells. S34F, serine-34 to phenylalanine substitution; wt wild-type.
(XLSX)

S2 Table Quantification of translation efficiency from polysome profiling and protein abundance from quantitative MS. Geometric means of the quantifications from both assays is shown. MS, mass spectrometry
(XLSX)

S3 Table Quantitative MS results in wt/wt and wt/S34F treated with control or NPM1 siRNA. Three replicates were run for each sample. Geometric means of the replicates are shown. MS, mass spectrometry; NPM1, Nucleophosmin 1; siRNA, small interfering RNA; S34F, serine-34 to phenylalanine substitution; wt, wild-type.
(XLSX)

S4 Table Co-occurring and mutually exclusive MDS and AML patient mutations obtained from the cBioPortal database. AML, Acute Myeloid Leukemia; MDS, Myelodysplastic Syndrome.
(XLSX)

S5 Table Quantification of translation efficiency from polysome profiling data using the polysome/monosome estimator in wt/wt, wt/S34F, and wt/S34F- cells. S34F, serine-34 to phenylalanine substitution; wt, wild-type.
(XLSX)

S1 Data Numerical data for figures. (XLSX)

S1 Raw Images Raw images for blots. (PDF)

## Abbreviations

AHA: azido-homoalanine
AML: Acute Myeloid Leukemia
ARF: Alternate Reading Frame
BCA: bicinchoninic acid
CCNA2: Cyclin A2
CD: cluster of differentiation
Col1a1: collagen, type I, alpha 1
CPM: counts per million
CrPV: Cricket Paralysis Virus
CHX: cycloheximide
CCNA2: Cyclin A2
*DDX*: DEAD-box helicase
*DHX*: DEAH-box helicase
EdU: 5-ethynyl-2ʹ-deoxyuridine
*EIF*: eukaryotic initiation factor
ER: estrogen receptor
FBS: fetal bovine serum
FC: fold change
FDR: false discovery rate
FUNCAT: fluorescent noncanonical amino acid tagging
FWER: family-wise error rate
*GAPDH*: glyceraldehyde-3-phosphate dehydrogenase
GFP: green fluorescent protein
GMP: granulocyte/macrophage progenitor
GO: Gene Ontology
GSEA: gene set enrichment analysis
HBEC: human bronchial epithelial cell
Hoxb8: Homeobox B8
IDH: isocitrate dehydrogenase
IPO7: Importin 7
IRES: Internal Ribosome Entry Site
ITS: internal transcribed spacer
*LAMTOR*: late endosomal/lysosomal adaptor, MAPK and MTOR activator
LAS1L: LAS1-like ribosome biogenesis factor
LUAD: Lung Adenocarcinoma
MDM2: mouse 3T3 cell double minute 2 proto-oncogene
MDS: Myelodysplastic Syndrome
MS: mass spectrometry
mSCF: murine stem cell factor
mTOR: mechanistic target of rapamycin kinase
NPM1: Nucleophosmin 1
OPP: O-propargyl-puromycin
p53: protein 53
RBM10: RNA binding motif protein 10
rDNA: ribosomal DNA
RIP: RNP immunoprecipitation
RIP-seq: RNP immunoprecipitation-Sequencing
RNAi: RNA interference
RNA-seq: RNA sequencing
*RPL*: ribosomal protein large subunit
*RPS*: ribosomal protein small subunit
*RPTOR*: regulatory associated protein of MTOR complex 1
rRNA: ribosomal RNA
RT-qPCR: reverse transcription–quantitative PCR
SF3B1: splicing factor 3 subunit 1
siRNA: small interfering RNA
SRSF2: serine and arginine rich splicing factor 2
S34F: serine-34 to phenylalanine substitution
TBTA: Tris[(1-benzyl-1H-1,2,3-triazol-4-yl)methyl]amine
TCEP: Tris(2-carboxyethyl)phosphine
TMT: tandem mass tag
*TSR2*: TSR2 ribosome maturation factor
U2AF1: U2 Small Nuclear RNA Auxiliary Factor 1
U2AF2: U2 Small Nuclear RNA Auxiliary Factor 2
WST-1: water-soluble tetrazolium-1
wt: wild-type
ZRSR2: zinc finger CCCH-type, RNA binding motif, and serine/arginine rich 2
5ʹ ETS: 5ʹ external transcribed spacer
