# Supplementary figures and images for: Ribosome biogenesis is a downstream effector of the oncogenic U2AF1-S34F mutation
Source: PLoS Biol. 2020 Nov 2;18(11):e3000920. doi: 10.1371/journal.pbio.3000920 (PMC7660540; doi:10.1371/journal.pbio.3000920)

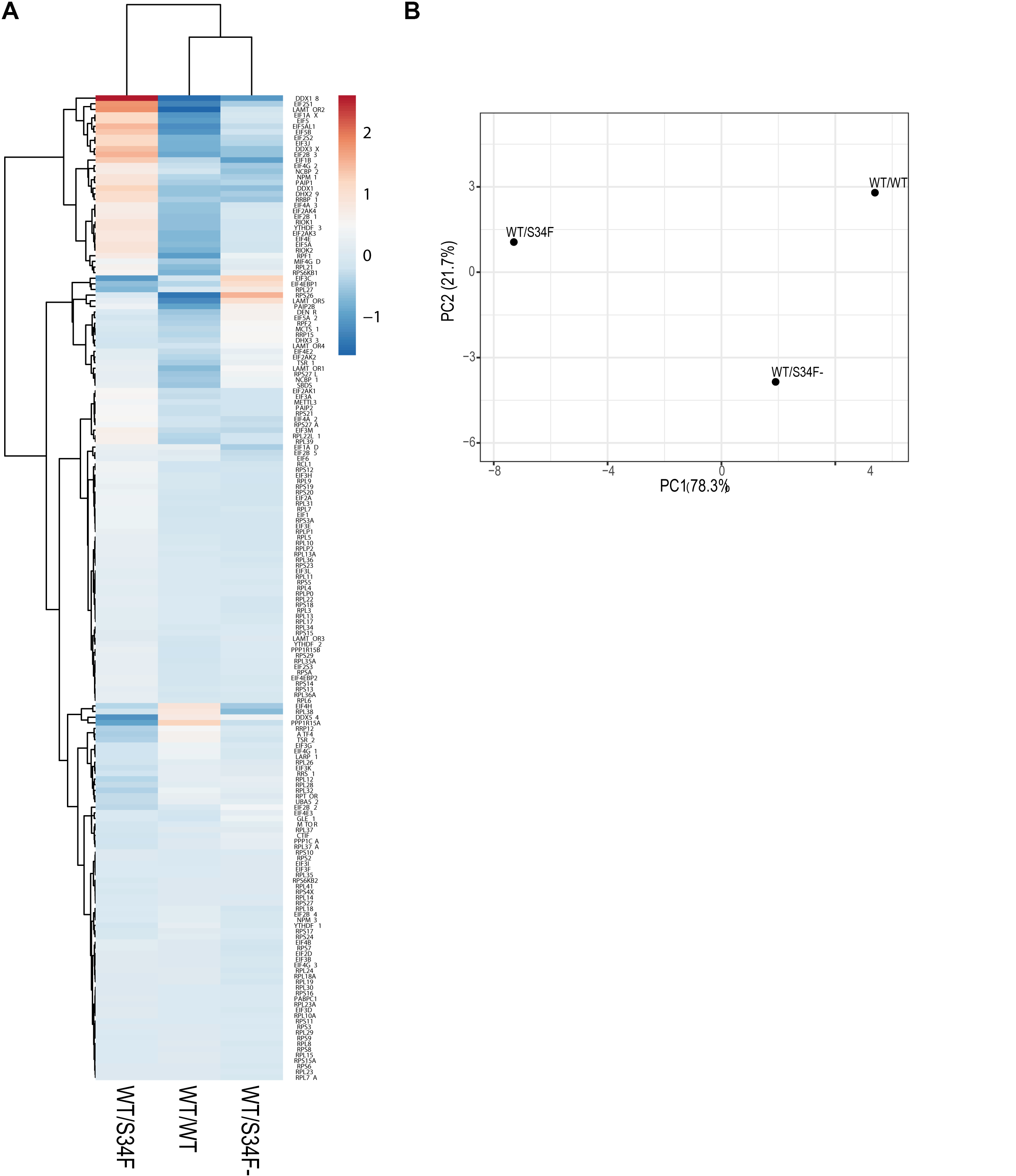

Supplement: S1 Fig — (A) Heat map and hierarchical clustering of the polysome/monosome estimator applied to RNA-seq from polysome fractions. The genes are the 175 translation initiation genes present in all 8 fractions of each sample (wt/wt, wt/S34F, wt/S34F-) cells (24 total measurements). Rows are centered; no scaling is applied to rows. Rows are clustered using Euclidean distance and Ward linkage. Columns are clustered using correlation distance and average linkage. 175 rows, 3 columns. (B) Principal component analysis of polysome/monosome estimator applied to RNA-seq from polysome fractions. No scaling is applied to rows; SVD with imputation is used to calculate principal components. X and Y axis show PC 1 and PC 2, which explain 78.3% and 21.7% of the total variance, respectively. The underlying data are in S5 Table. PC, principal component; RNA-seq, RNA sequencing; SVD, singular value decomposition; S34F, serine-34 to phenylalanine substitution; wt, wild-type. (TIF) [file pbio.3000920.s001.tif]

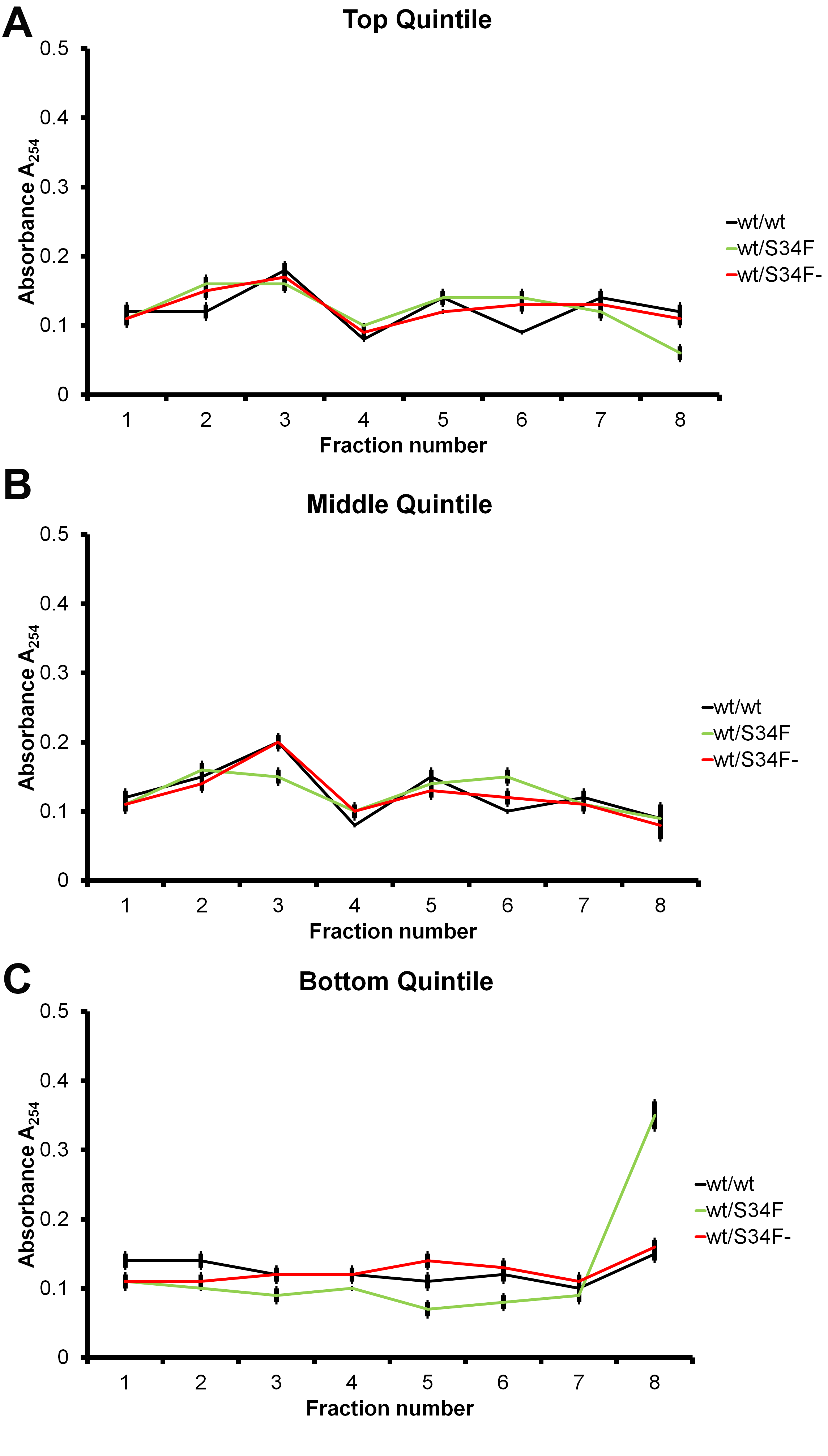

Supplement: S2 Fig — (A) Polysome profile for the top quintile mRNAs. (B) Polysome profile for the middle quintile mRNAs. (C) Polysome profile for the bottom quintile mRNAs. The underlying data are in S1 Data. (TIF) [file pbio.3000920.s002.tif]

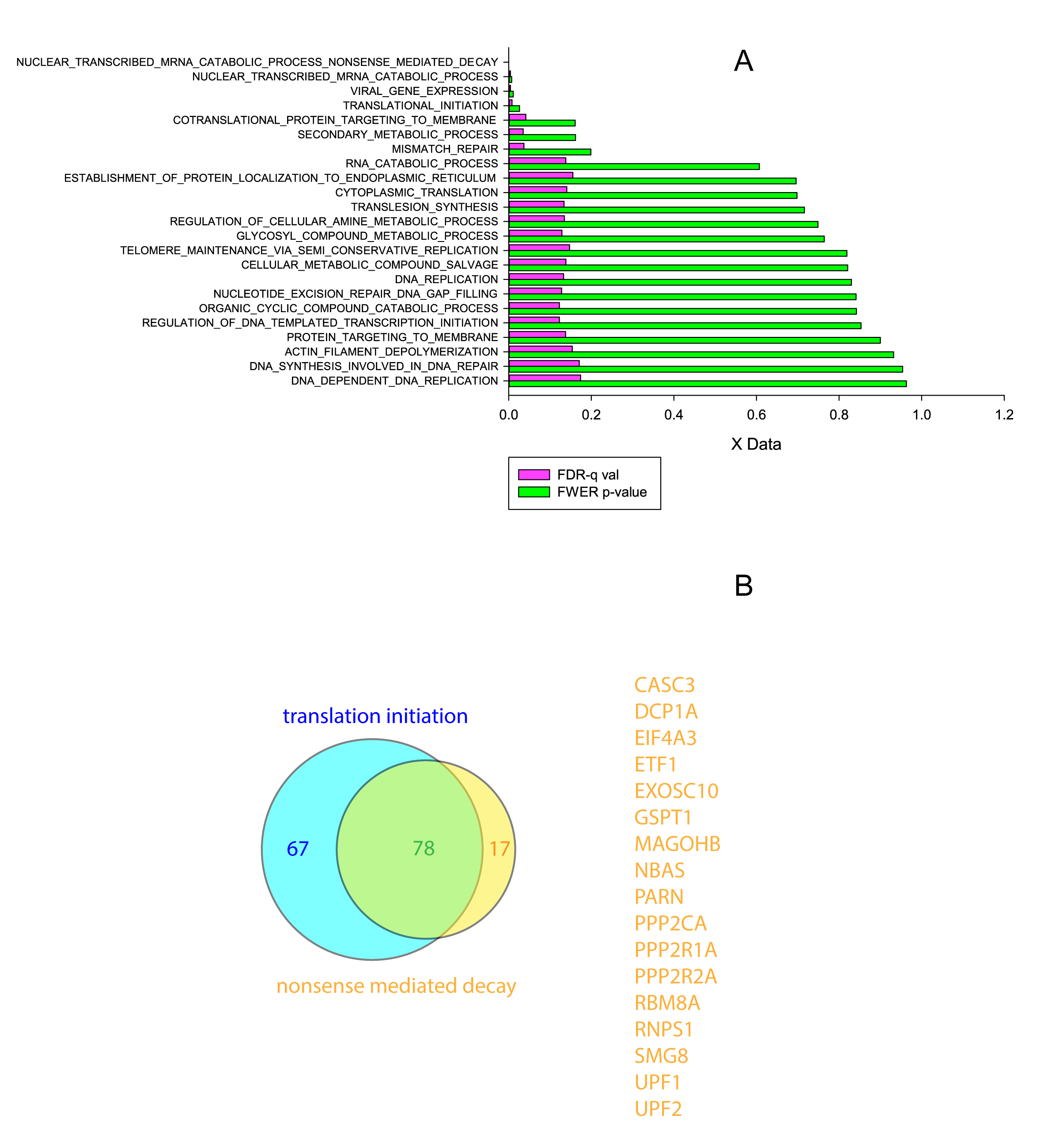

Supplement: S3 Fig — (A) The top 4 GO categories show similar FDR and FWER and contain many of the same genes. The underlying data are in S2 Table. (B) The overlap between the GO categories of “nuclear transcribed mRNA catabolic process nonsense mediated decay” and “translation initiation” is shown as a Venn diagram. The 17 genes that are present in “nuclear transcribed mRNA catabolic process nonsense mediated decay,” but not “translation initiation,” are listed. FDR, false discovery rate; FWER, family-wise error rate; GO, Gene Ontology; GSEA, gene set enrichment analysis; MS, mass spectrometry (TIF) [file pbio.3000920.s003.tif]

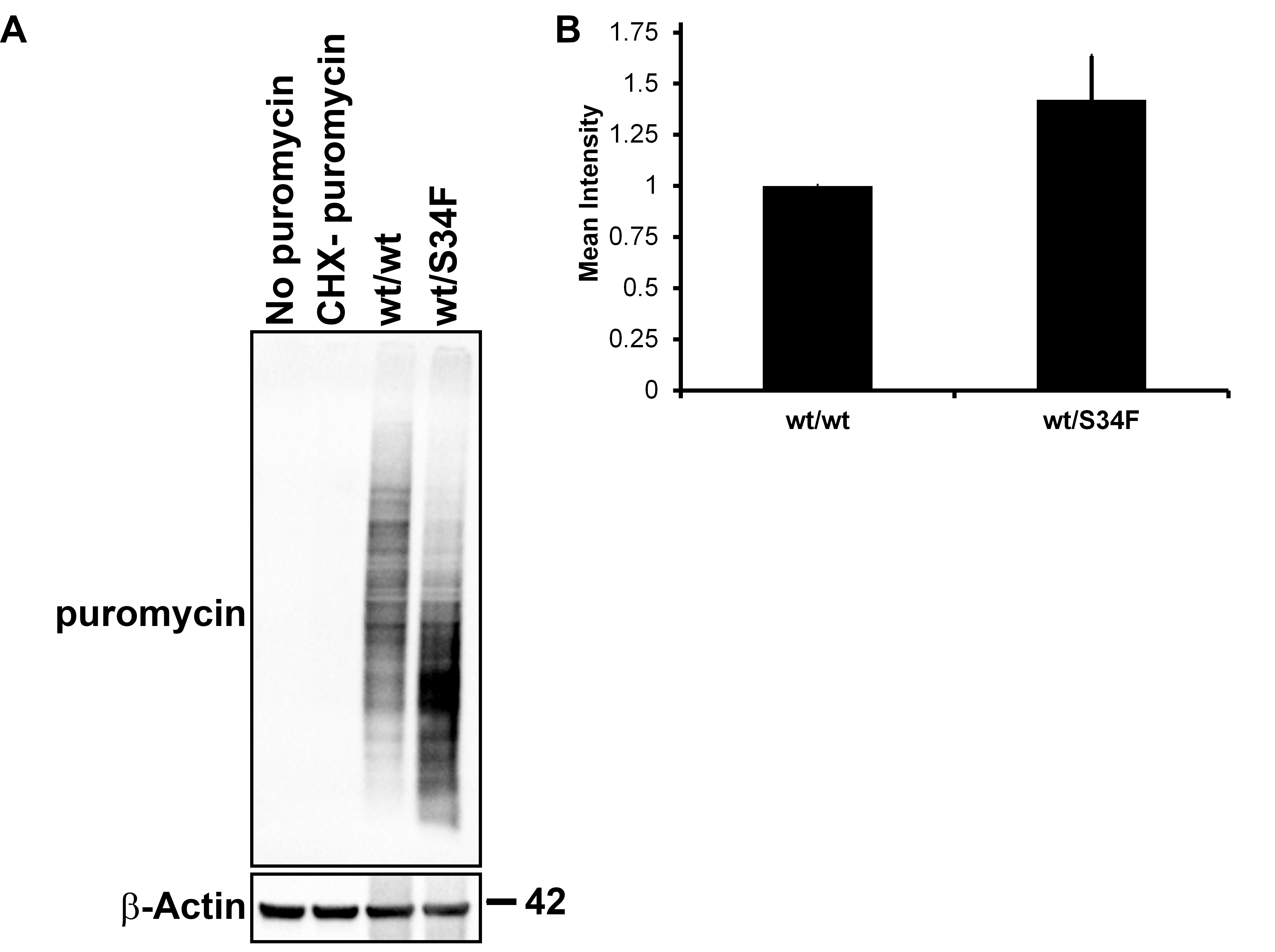

Supplement: S4 Fig — (A) Representative western blot of puromycin-labeled nascent polypeptides. (B) Quantification of the signal intensity. Each bar represents the average and standard error of 3 independent experiments. The underlying data are in S1 Data. CHX, cycloheximide; S34F, serine-34 to phenylalanine substitution; wt, wild-type. (TIF) [file pbio.3000920.s004.tif]

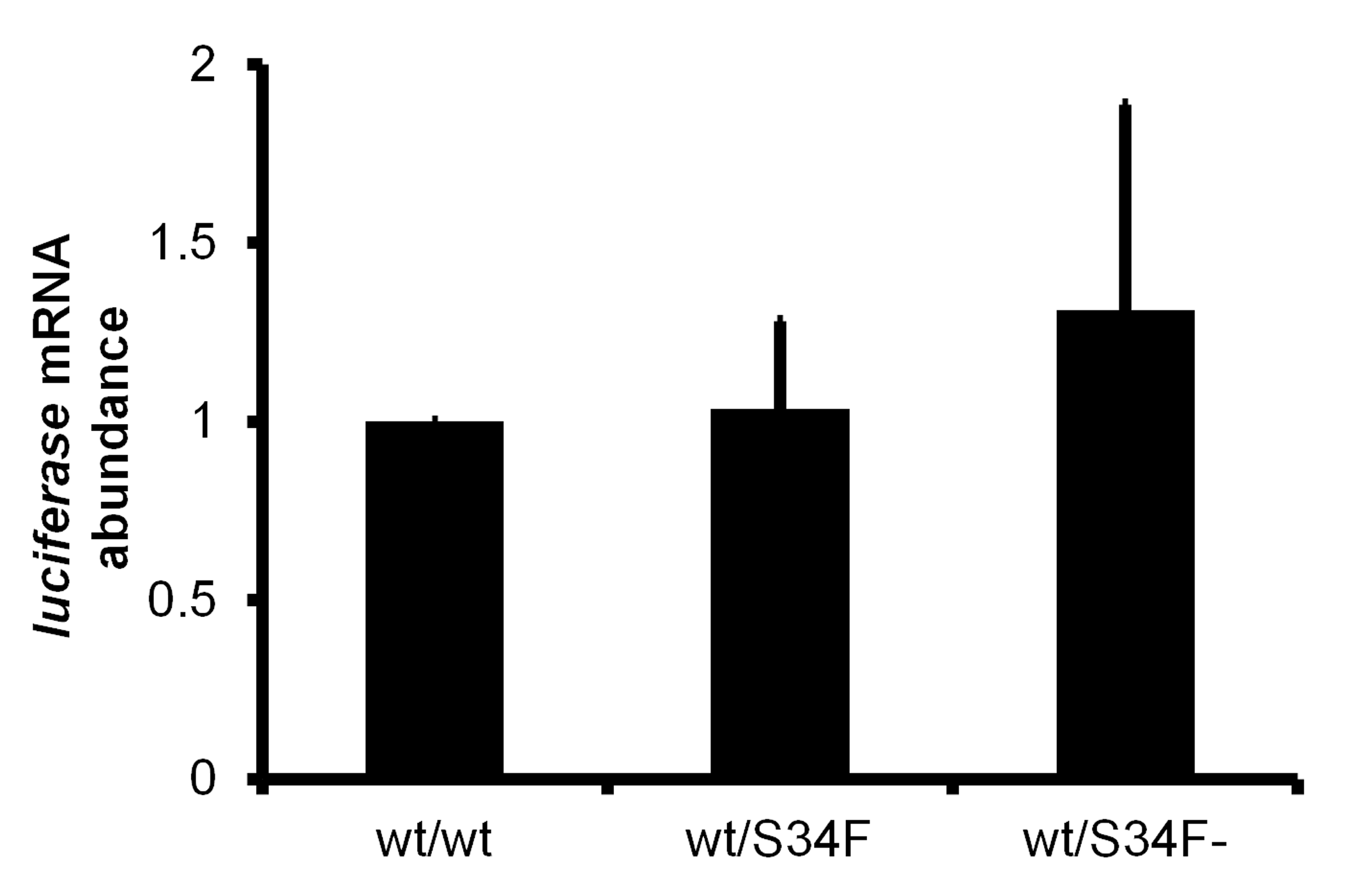

Supplement: S5 Fig — The NPM1-luciferase mRNA levels were normalized to TUBA1A mRNA. Each bar represents the average and standard error of 3 independent experiments. The underlying data are in S1 Data. NPM1, Nucleophosmin 1; RT-PCR, reverse transcription–quantitative PCR; S34F, serine-34 to phenylalanine substitution; TUBA1A, tubulin alpha 1a; wt, wild-type. (TIF) [file pbio.3000920.s005.tif]

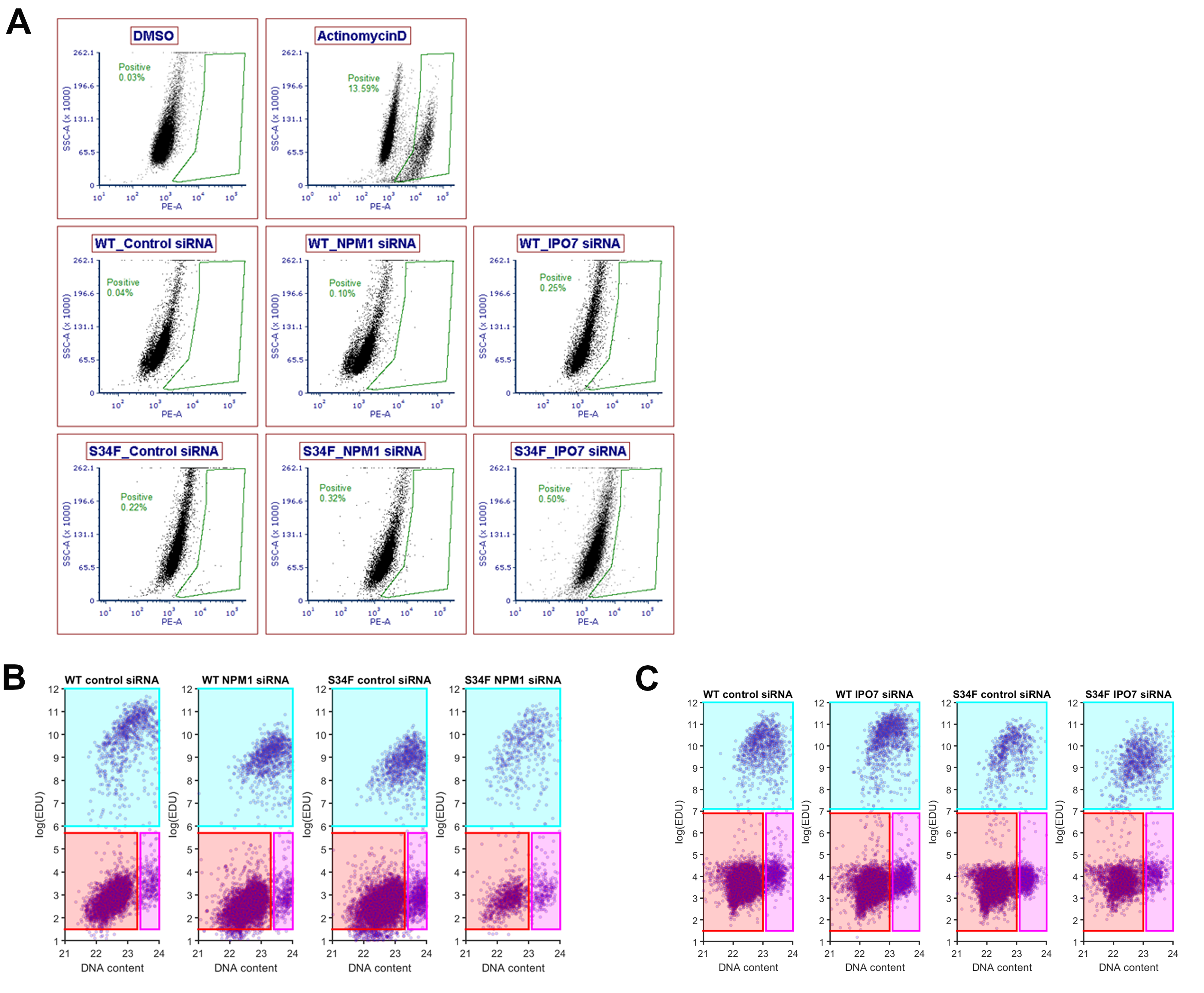

Supplement: S6 Fig — (A) Gating strategy from a representative cleaved Caspase-3 experiment. (B) Gating strategy from a representative EdU labeling experiment for NPM1 siRNA. (C) Gating strategy from a representative EdU labeling experiment for IPO7 siRNA. DMSO, dimethyl sulfoxide; EdU, 5-ethynyl-2ʹ-deoxyuridine; IPO7, Importin 7; NPM1, Nucleophosmin 1; PE-A, Phycoerythrin-Area; siRNA, small interfering RNA; SSC-A, side scatter-Area (TIF) [file pbio.3000920.s006.tif]

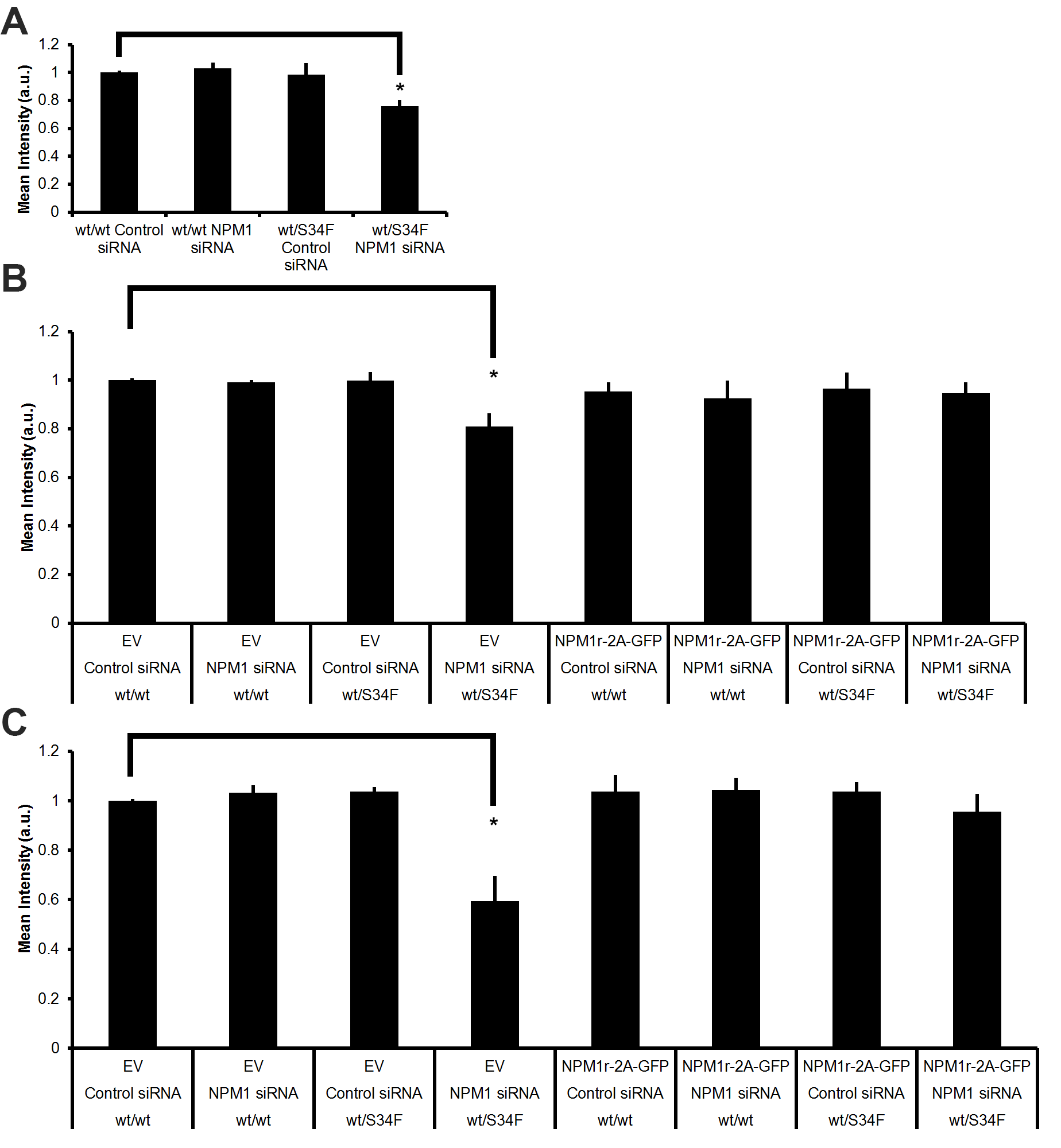

Supplement: S7 Fig — (A) Quantification of the ITS2 northern blot showing wt/wt and wt/S34F cells treated with control or NPM1 siRNA. Each bar represents the average and standard error of 3 independent experiments. *p < 0.05 using a paired 2-sided t test. (B) Quantification of the ITS2 northern blot showing wt/wt and wt/S34F cells that overexpress an empty vector or the NPM1r-2A-GFP reporter and were treated with control or NPM1 siRNA. Each bar represents the average and standard error of 3 independent experiments. *p < 0.05 using a paired 2-sided t test. (C) Quantification of the 5ʹ ETS northern blot showing wt/wt and wt/S34F cells that overexpress an empty vector or the NPM1r-2A-GFP reporter and were treated with control or NPM1 siRNA. Each bar represents the average and standard error of 3 independent experiments. *p < 0.05 using a paired 2-sided t test. The underlying data are in S1 Data. A.U., auxiliary unit; EV, empty vector; GFP, green fluorescent protein; ITS2, internal transcribed spacer 2; NPM1, Nucleophosmin 1; siRNA, small interfering RNA; S34F, serine-34 to phenylalanine substitution; wt, wild-type; 5ʹ ETS, 5ʹ external transcribed spacer (TIF) [file pbio.3000920.s007.tif]
