# Supplementary material for: Ribosome biogenesis is a downstream effector of the oncogenic U2AF1-S34F mutation
Source: PLoS Biol. 2020 Nov 2;18(11):e3000920. doi: 10.1371/journal.pbio.3000920 (PMC7660540; doi:10.1371/journal.pbio.3000920)

Fig. 2C:

NPM1:

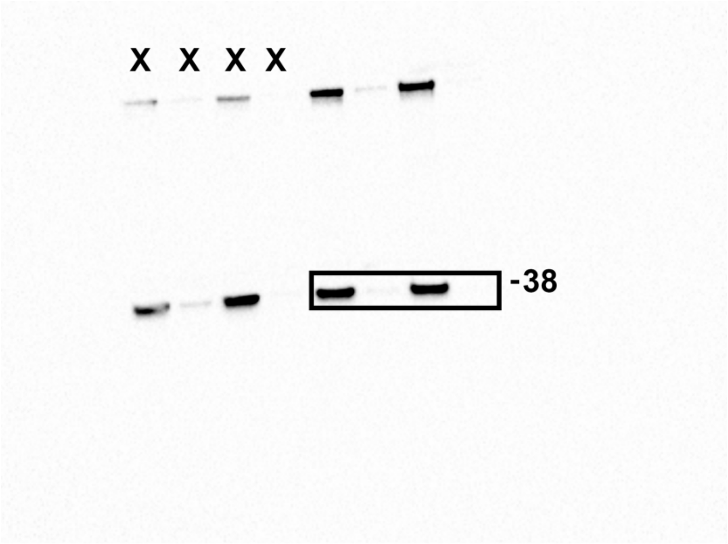

Actin:

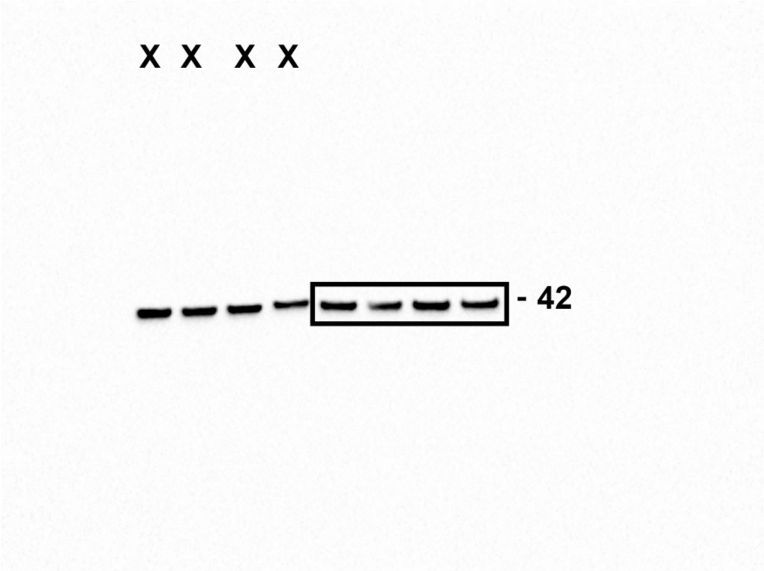

Fig 2F:

NPM1:

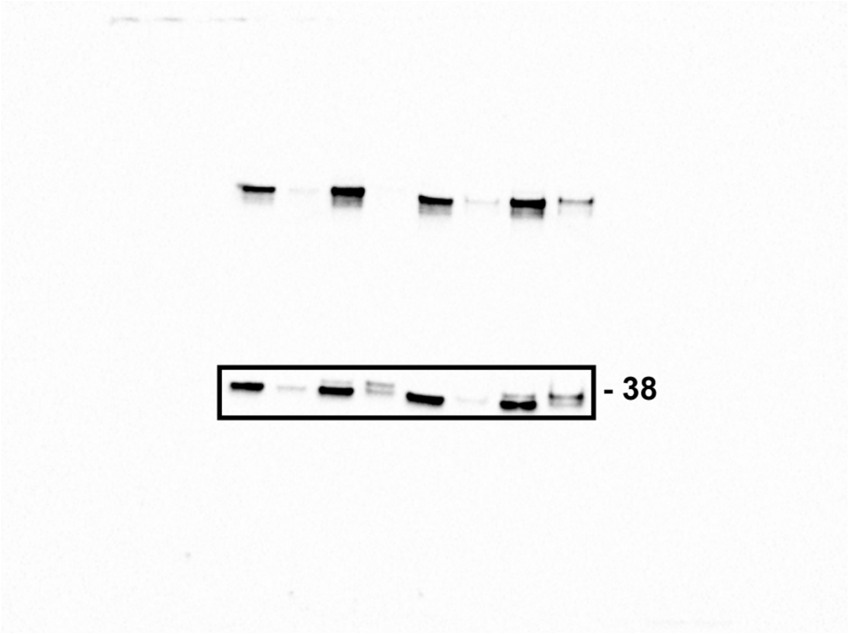

Actin:

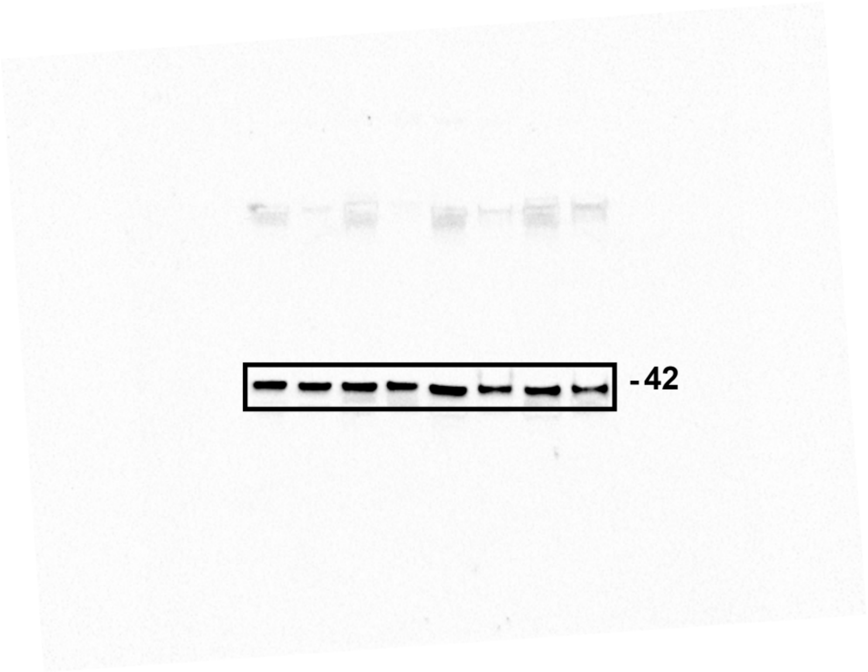

Fig 2H:

IPO7:

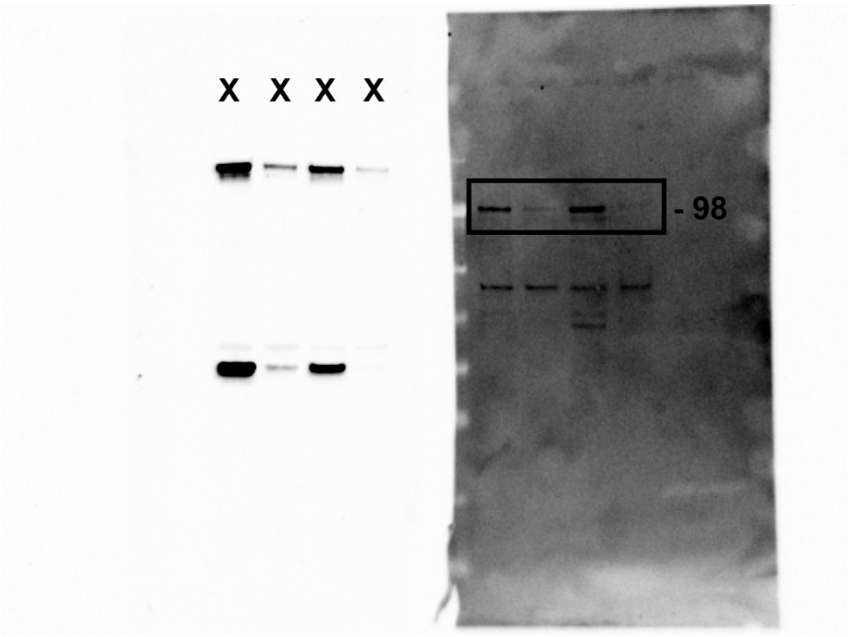

Actin:

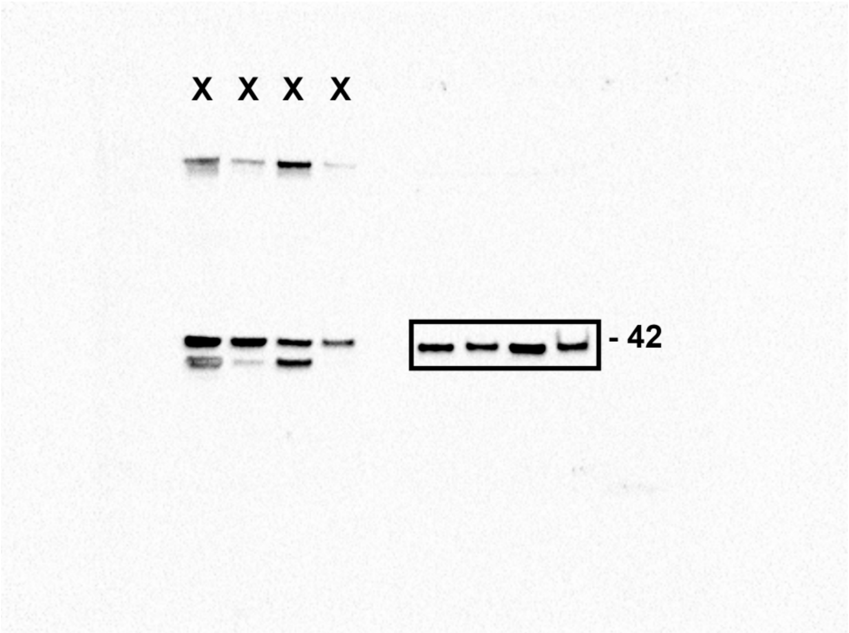

Fig. 2J:

RBM10:

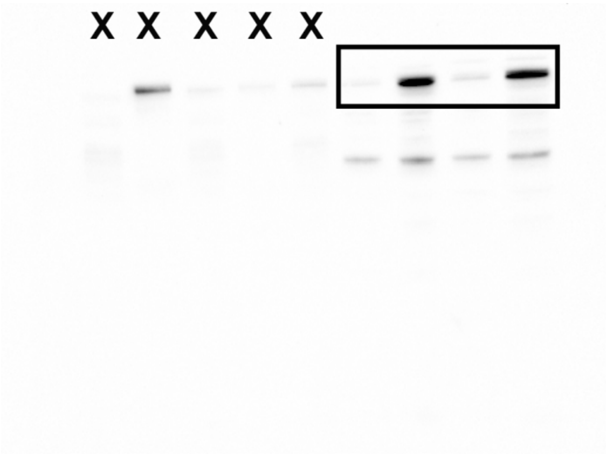

Tubulin:

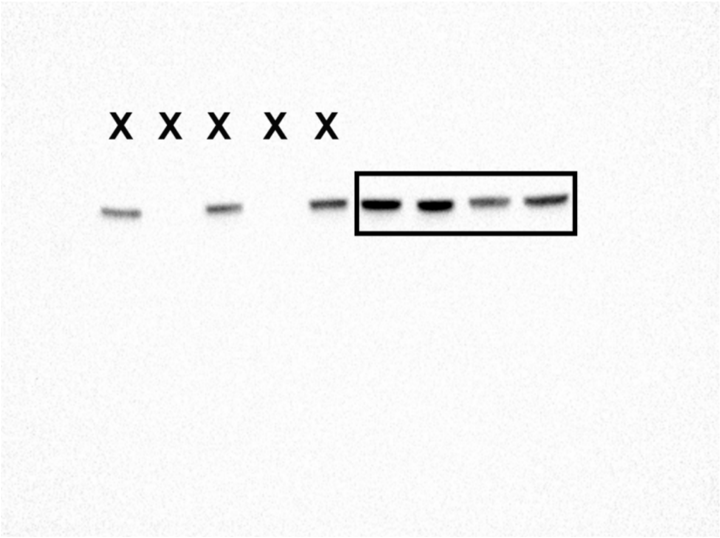

Fig 3A:

NPM1:

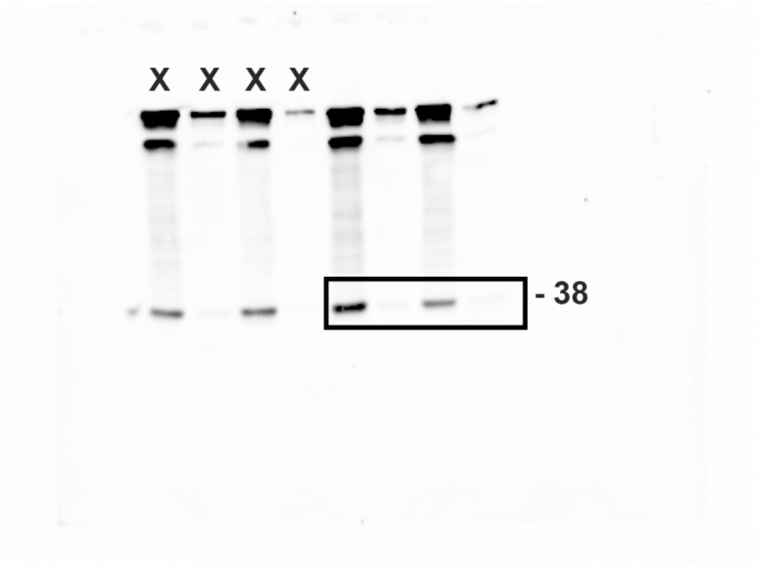

Tubulin:

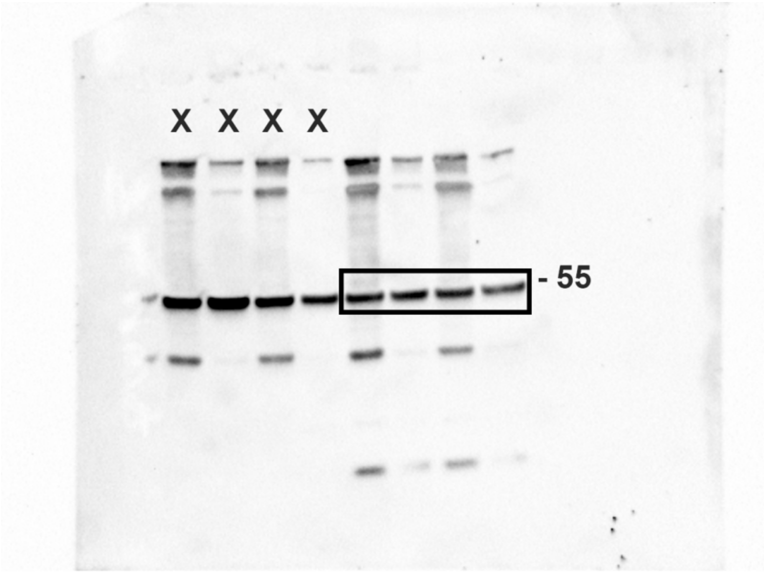

p53:

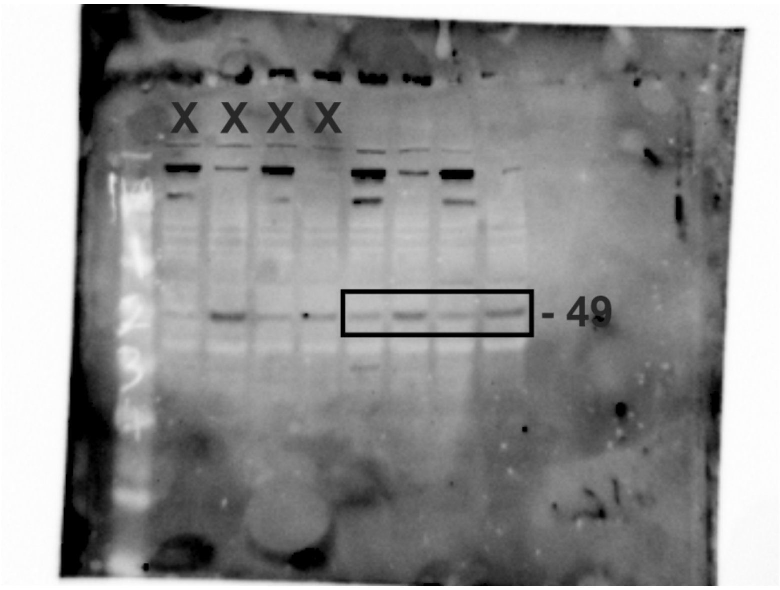

Fig. 3D:

NPM1:

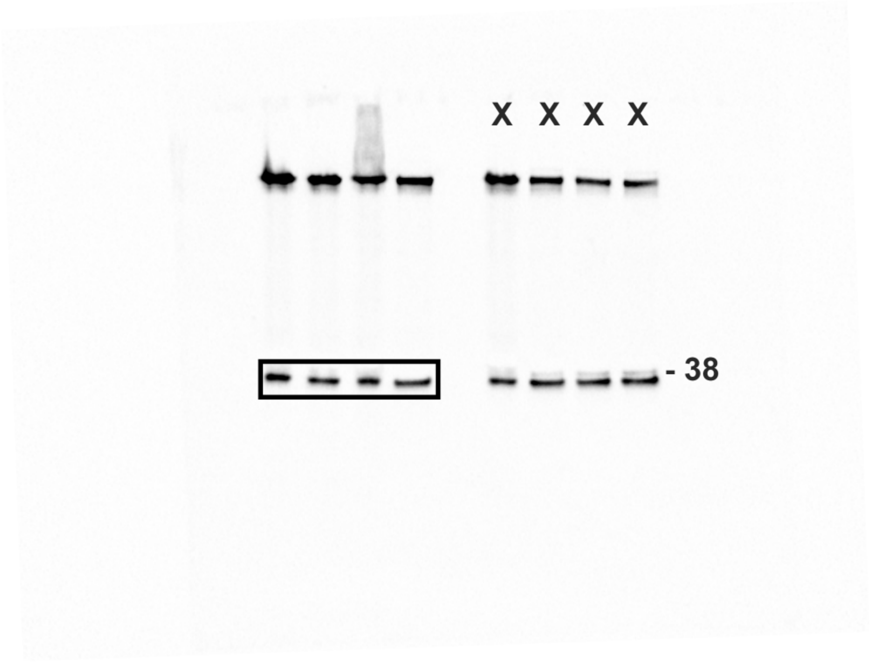

Actin:

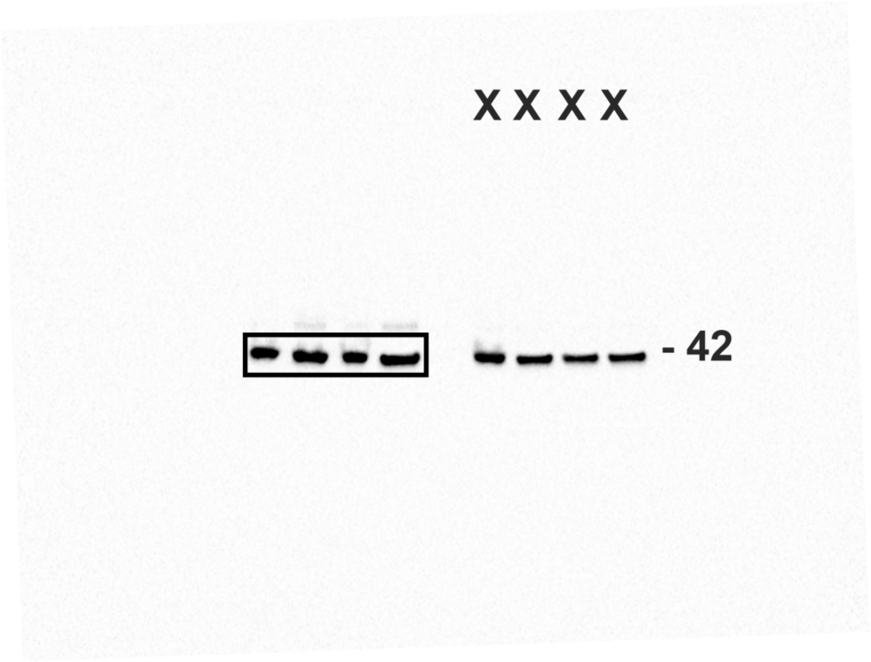

p53:

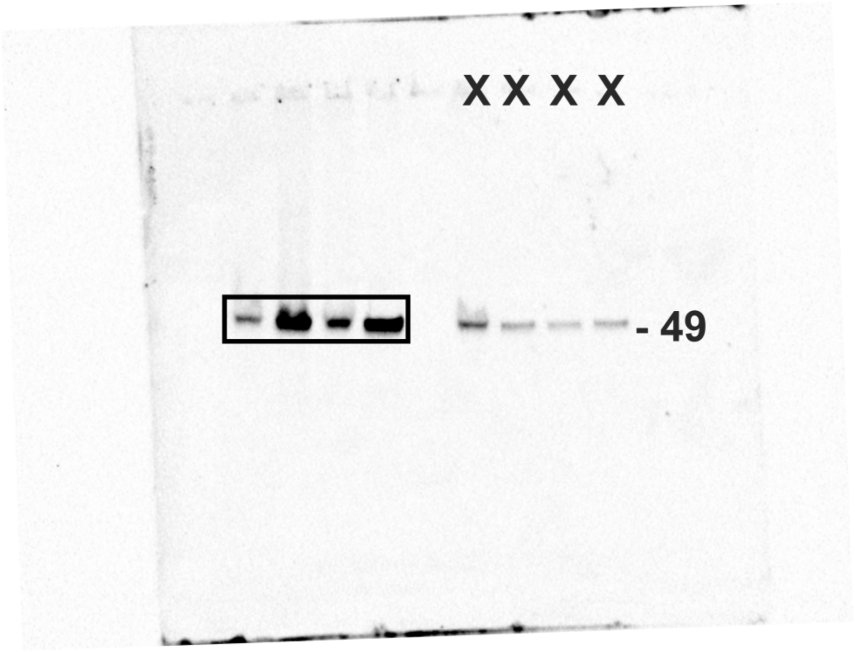

Fig. 4E:

NPM1:

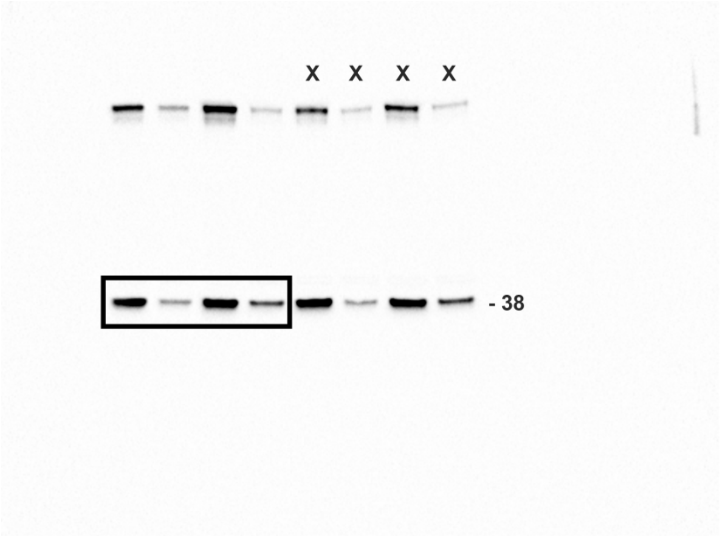

Actin:

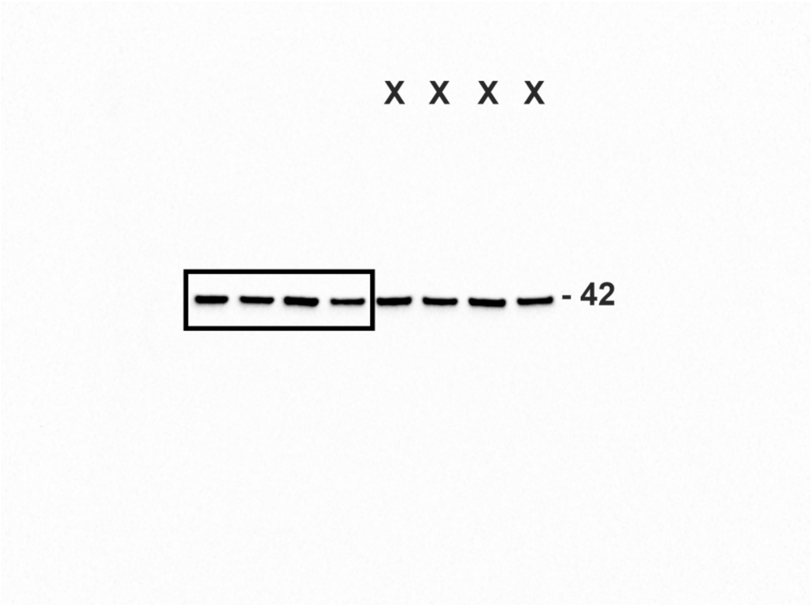

Cyclin A2:

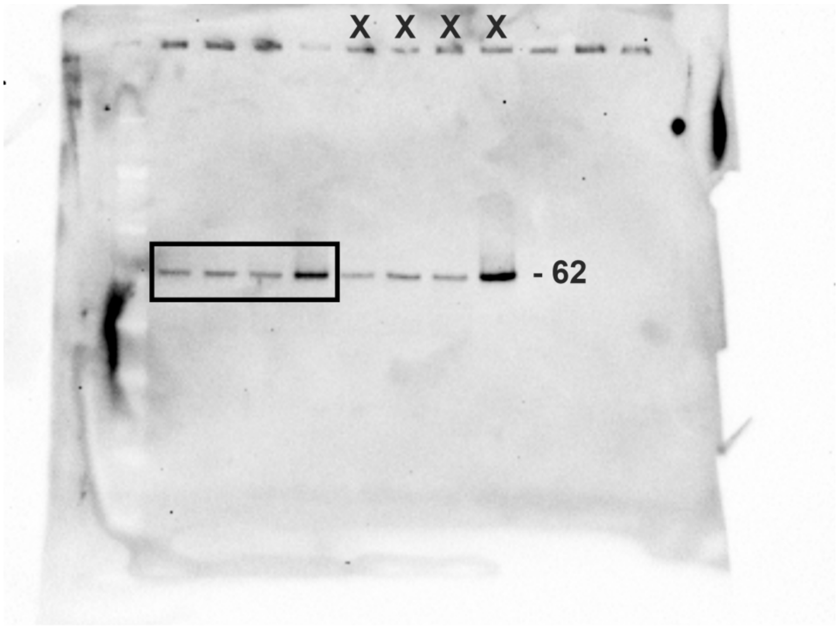

Fig. 4F:

IPO7:

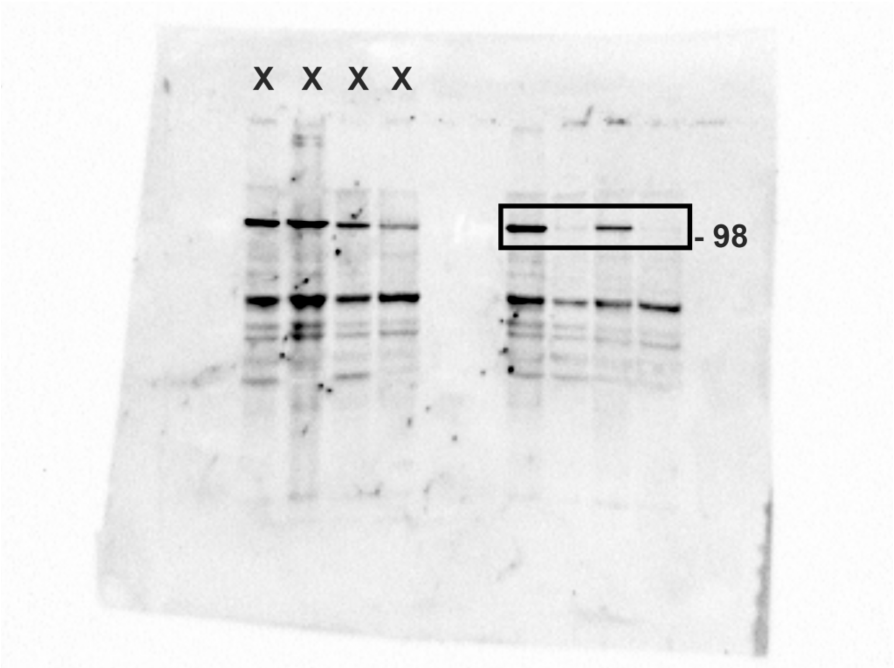

Actin:

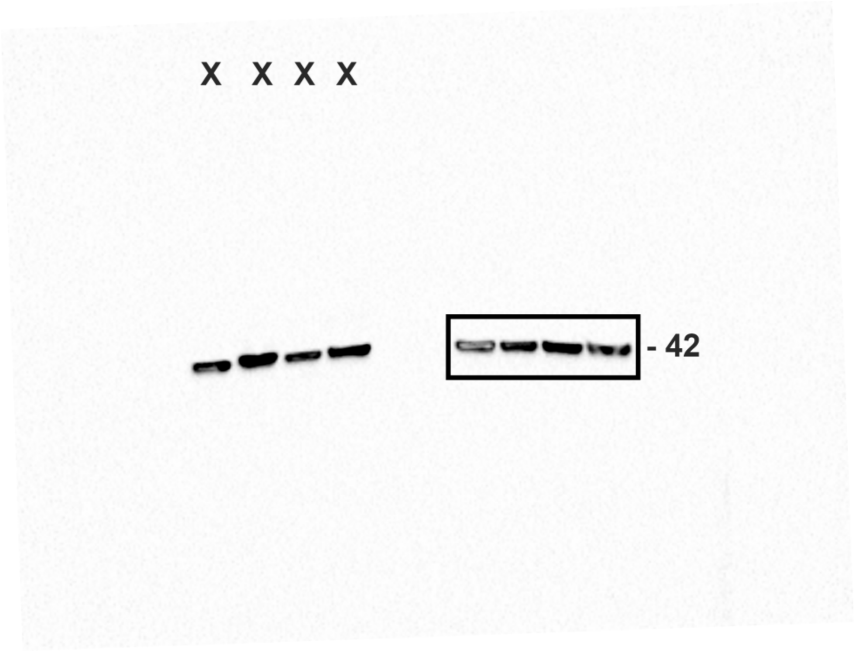

Cyclin A2:

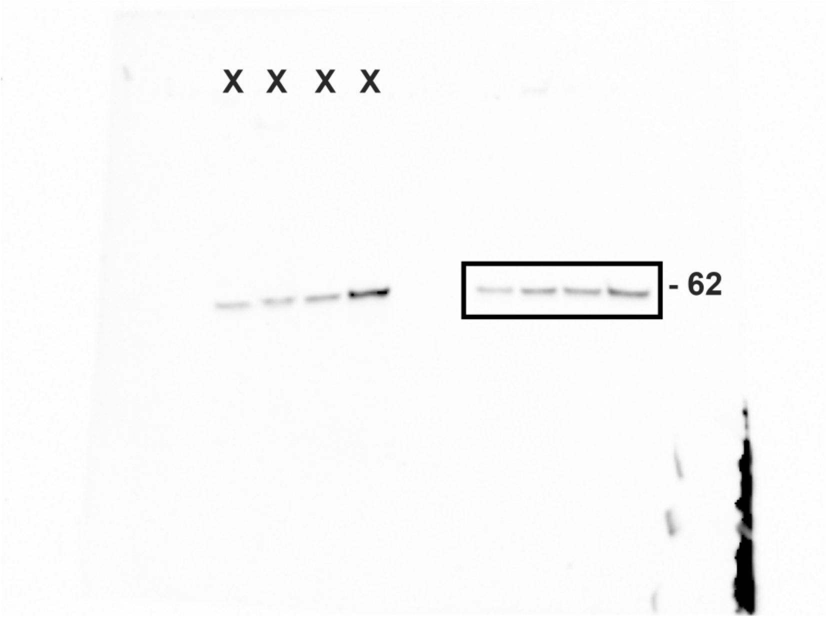

Fig. 5B:

ITS2:

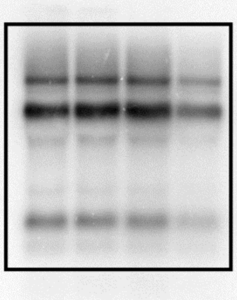

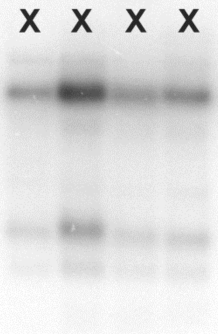
x x x x

GAPDH:

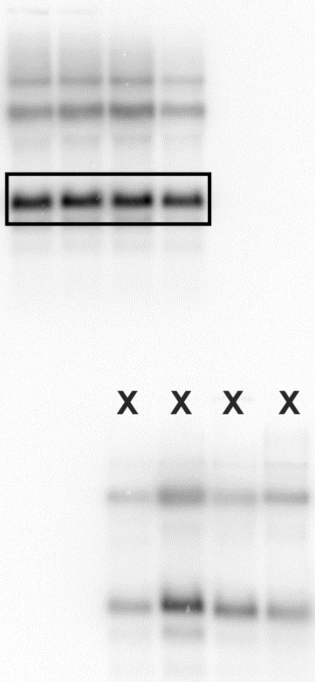
X X X X

Ethidium Bromide (zoom-in high exposure uncropped image of only top half of gel):

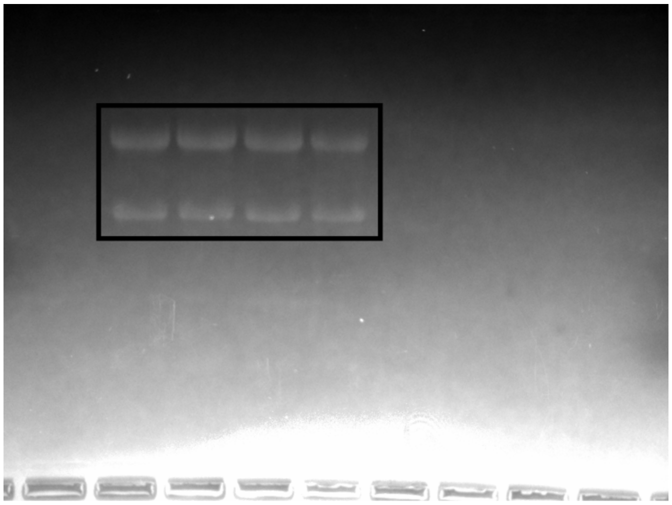

Ethidium bromide (zoom-out low exposure uncropped image of whole gel):

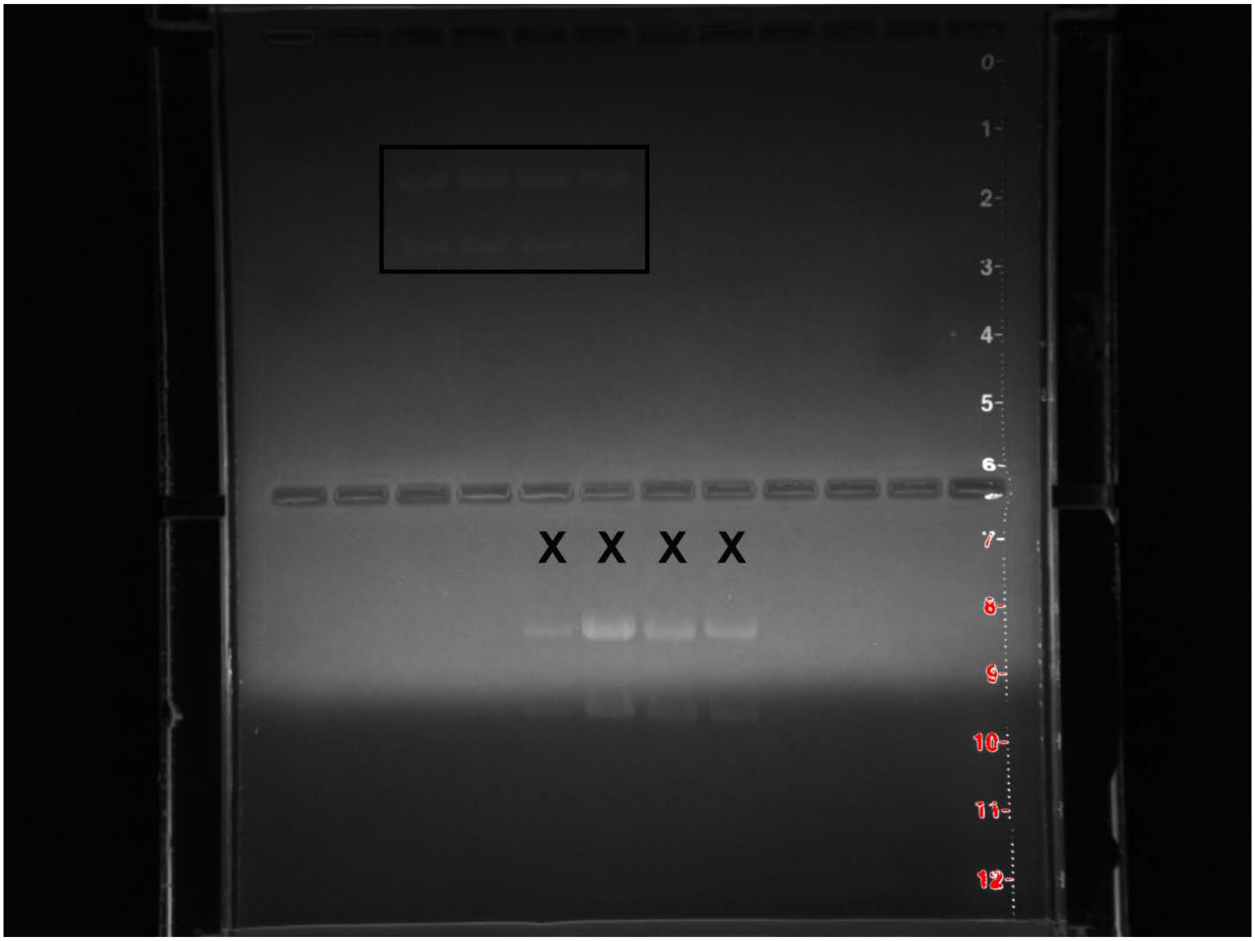

Fig. 5C:

ITS2:

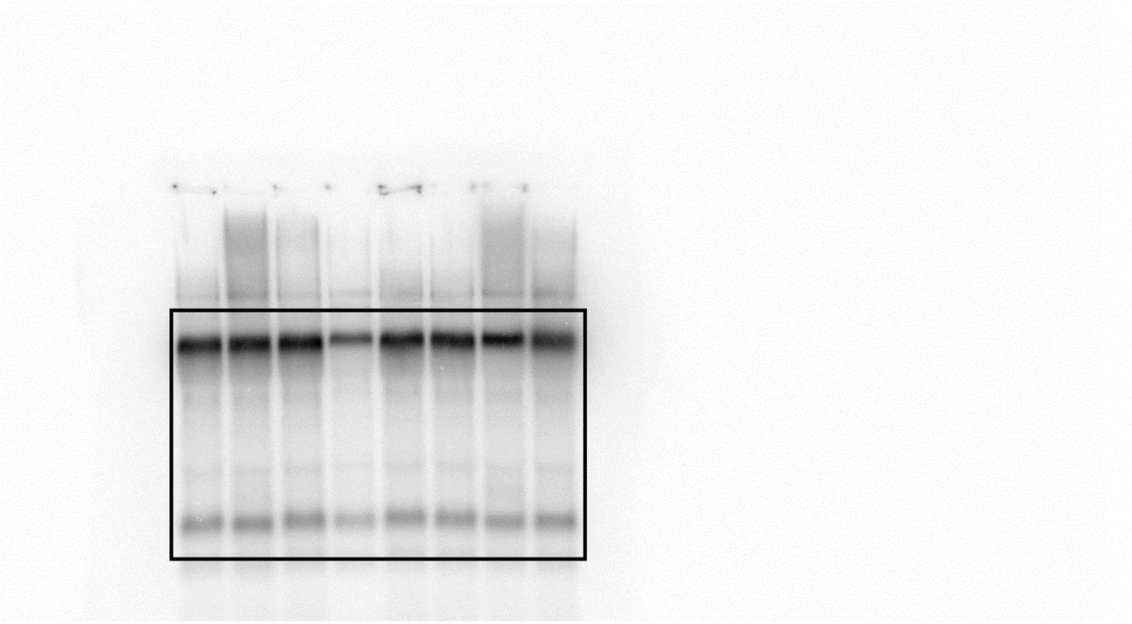

GAPDH:

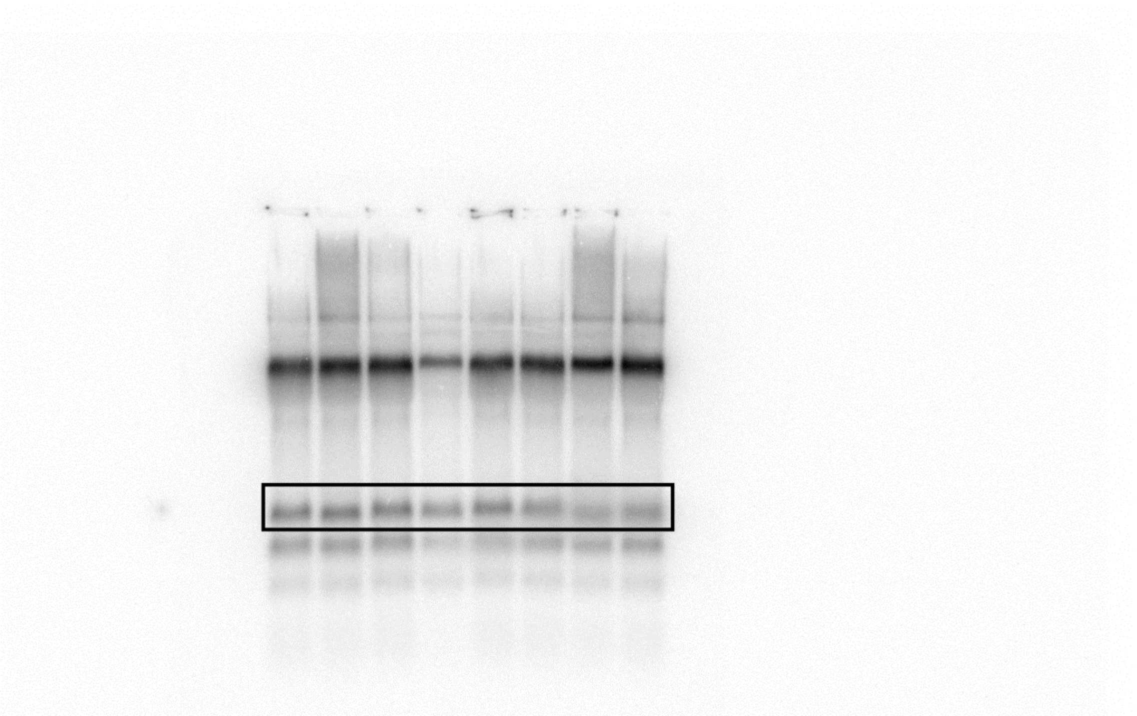

Ethidium Bromide:

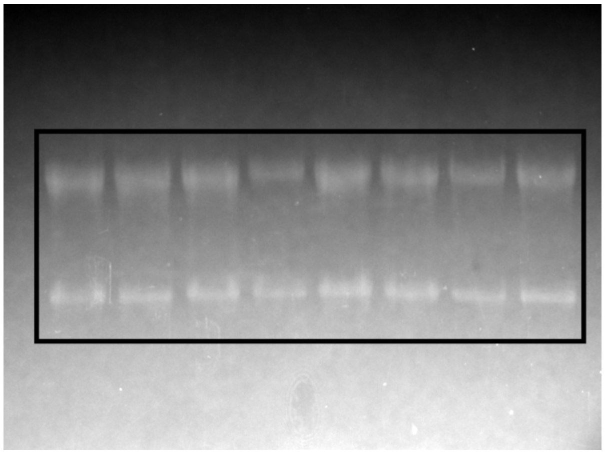

Fig. 5D:

5’-ETS:

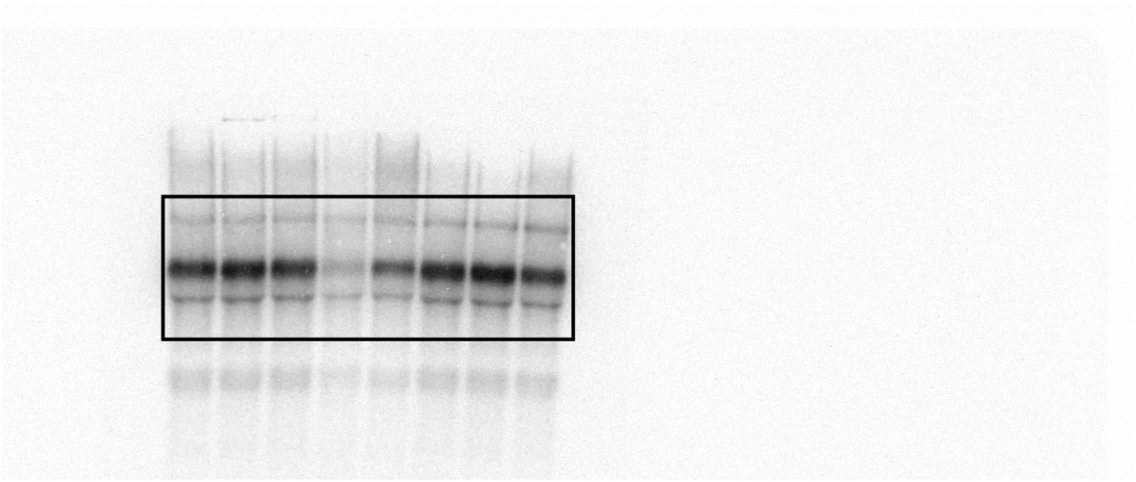

GAPDH:

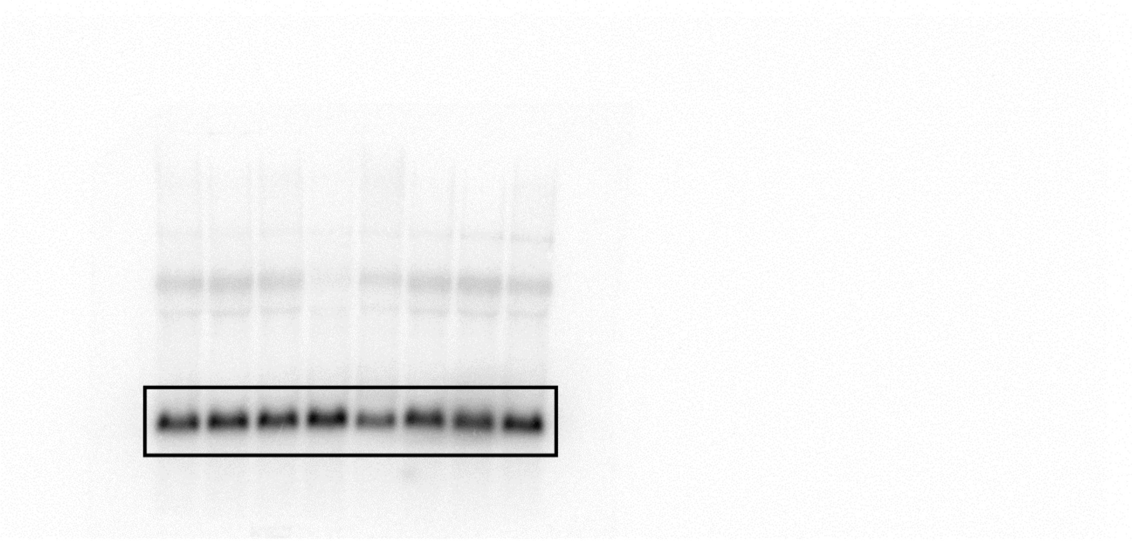

Ethidium Bromide:

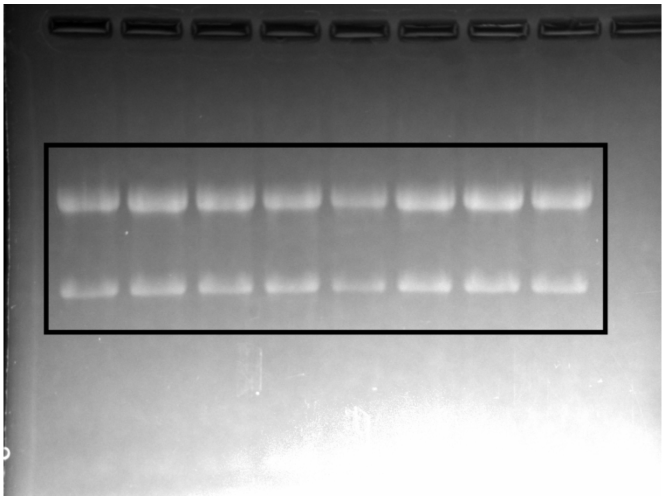

S4 Fig:

puromycin

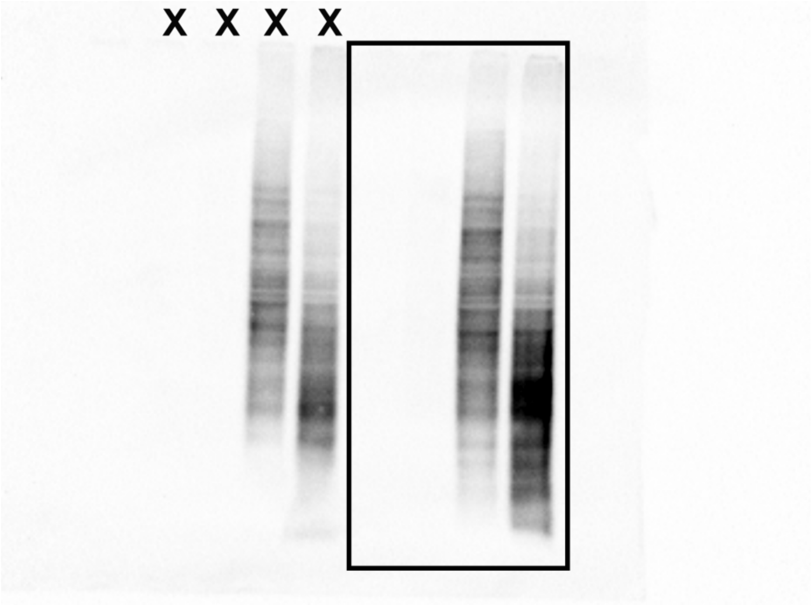

Actin

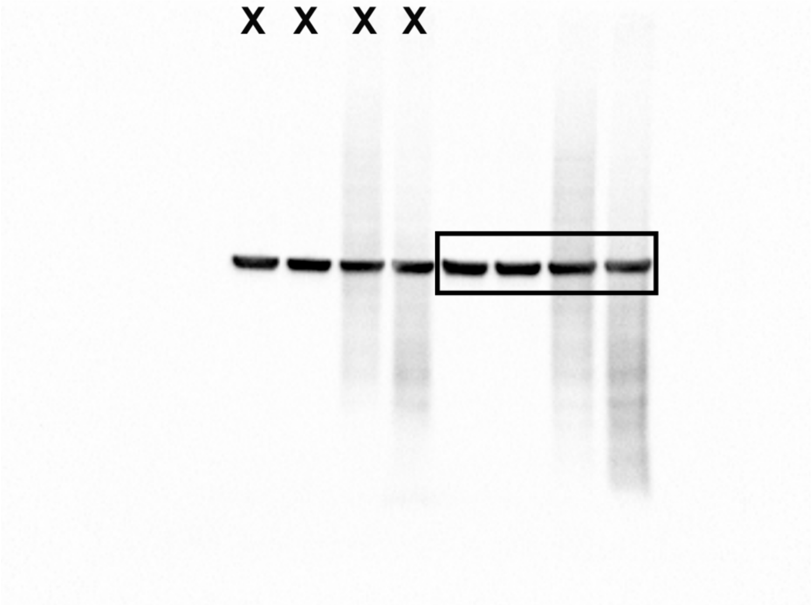

Supplement: S1 Raw Images — (PDF) [file pbio.3000920.s014.pdf]
